# Exploration of Uncharted Regions of the Protein Universe

**DOI:** 10.1371/journal.pbio.1000205

**Published:** 2009-09-29

**Authors:** Lukasz Jaroszewski, Zhanwen Li, S. Sri Krishna, Constantina Bakolitsa, John Wooley, Ashley M. Deacon, Ian A. Wilson, Adam Godzik

**Affiliations:** 1 Joint Center for Structural Genomics, Bioinformatics Core, Burnham Institute for Medical Research, La Jolla, California, United States of America; 2 Joint Center for Molecular Modeling, Burnham Institute for Medical Research, La Jolla, California, United States of America; 3 Joint Center for Structural Genomics, Bioinformatics Core, Center for Research in Biological Systems, University of California San Diego, La Jolla, California, United States of America; 4 Joint Center for Structural Genomics, Structure Determination Core, Stanford Synchrotron Radiation Lightsource, SLAC National Accelerator Laboratory, Menlo Park, California, United States of America; 5 Joint Center for Structural Genomics, The Scripps Research Institute, La Jolla, California, United States of America; MRC Laboratory of Molecular Biology, United Kingdom

## Abstract

The genome projects have unearthed an enormous diversity of genes of unknown function that are still awaiting biological and biochemical characterization. These genes, as most others, can be grouped into families based on sequence similarity. The PFAM database currently contains over 2,200 such families, referred to as domains of unknown function (DUF). In a coordinated effort, the four large-scale centers of the NIH Protein Structure Initiative have determined the first three-dimensional structures for more than 250 of these DUF families. Analysis of the first 248 reveals that about two thirds of the DUF families likely represent very divergent branches of already known and well-characterized families, which allows hypotheses to be formulated about their biological function. The remainder can be formally categorized as new folds, although about one third of these show significant substructure similarity to previously characterized folds. These results infer that, despite the enormous increase in the number and the diversity of new genes being uncovered, the fold space of the proteins they encode is gradually becoming saturated. The previously unexplored sectors of the protein universe appear to be primarily shaped by extreme diversification of known protein families, which then enables organisms to evolve new functions and adapt to particular niches and habitats. Notwithstanding, these DUF families still constitute the richest source for discovery of the remaining protein folds and topologies.

## Abstract 2

Determination of first protein structures, from hundreds of families of unknown function, have shown that divergence, rather than novelty, is the dominant force that shapes the evolution of the protein universe.

## Author Summary

More than 40% of known proteins lack any annotation within public databases and are usually referred to as hypothetical proteins despite most of them being real and many being evolutionarily conserved and thus expected to play important biological roles. Determination of the three-dimensional structures of representatives of more than 240 families of protein domains of unknown function by the Protein Structure Initiative has provided a unique sample of regions of the protein universe that, until this systematic effort, were completely uncharacterized. Analysis of these structures reveals that most of the 240 families can be considered as remote homologs of already known protein families. Such distant evolutionary links can sometimes be predicted by current state-of-the-art sequence comparison tools, but structural analysis has led to the first hypotheses about biological functions for many of these uncharacterized proteins, and serves as a starting point for experimental studies. The rapid pace of discovery of such relationships appears to suggest that the protein universe is made up of a relatively small and stable number of ‘extended neighborhoods’ that bring together distantly related protein families. Thus, the vast uncharacterized part of protein universe, called by some “the dark matter of protein space”, may consist mainly of highly divergent homologs. Continued structural characterization of these previously under-investigated regions of the protein universe should further help unravel the patterns and rules that led to such divergence in the evolution of protein structure and function.

## Introduction

The sequences of several millions of proteins are currently known and this number is growing ever more rapidly as a result of the relentless efficiency of genomic and metagenomic sequencing projects. Around 30%–40% of these gene products are classified as so-called “hypothetical proteins.” This term is somewhat of a misnomer, but is the accepted way of indicating that no information is available about them other than the translated nucleotide sequence. It is interesting to note that this group of proteins persists despite years of annotation efforts in the genome sequencing projects. “Hypothetical proteins” are not merely artifacts, and many have been validated as gene products in function-based, genome-scale surveys, such as essentiality analysis [1],[2], disease association studies [3]–[5], genome-wide DNA expression arrays [6]–[8], cDNA and proteomics-based environmental surveys [9]–[12]. They then are bona fide proteins that simply have not yet been the focus of any detailed study. The importance of such “conserved hypotheticals” has been discussed many times in the literature [13] and proposed as an important subject area for further studies: “experimental characterization of […] ‘conserved hypothetical’ proteins is expected to reveal new, crucial aspects of microbial biology and could also lead to better functional prediction for medically relevant human homologs”[14]. We can expect that most of the yet undiscovered functionality of these families will represent novel chemistry, novel biochemical pathways, alternative solutions to known reactions, or new regulatory mechanisms. The fact that they are usually overlooked or even omitted from many studies may introduce significant biases in “-omics” analyses [15]. Thus, the NIH Protein Structure Initiative (PSI; http://www.nigms.nih.gov/Initiatives/PSI/) has made a concerted and systematic effort to explore these uncharted regions of the protein universe as a means to uncover new insights into the evolution and diversity of protein structure and function.

Protein space can be dissected and organized by grouping proteins into families of homologs, based on inferred evolutionary and functional relationships. Many specialized resources [16]–[19] have been developed to provide information on protein families. All of these sources paint a similar picture of the protein universe, with only some quantitative differences that arise from use of different protocols and definitions of protein families. One of the oldest and best known such resource, the PFAM database [20] (http://pfam.janelia.org/), in its 23^rd^ release, lists over 10,000 protein families that cover around 70% of an average genome. The number of protein families listed by PFAM and other resources increase over time; for instance, 5 y ago PFAM listed only 5,000 families. Part of this increase can be accounted for by more rigorous analysis of the existing data, but the rapidly increasing number of known protein sequences is the main factor driving the apparent growth in the number of protein families. One of the most interesting questions in biology concerns the implications of this growth—do we expect that the number of protein families grows linearly with the number of known sequences, or at some point, does it start to saturate? Results from the analysis of metagenomics open reading frames (ORFs) [21], presented in this journal 2 y ago, seemed to suggest that we are still in the linear phase of growth of the number of protein families, but as we will show here, the picture is different when we look at the higher level of organization of the protein universe.

Protein families are most commonly defined by sequence similarity, as it represents the most obvious trace of an evolutionary relationship between proteins. However, as our ability to recognize sequence similarity between proteins has progressed from simple residue-by-residue comparisons measured by mutation matrices [22] to sequence profiles [23], position-specific mutation matrices [24] or Hidden Markov Models (HMM) [25],[26], to comparisons between such profiles [27] or between the HMM [28], it has become eminently clear that statistically significant sequence similarity between proteins may extend far beyond the intuitive definition based on sequence identity. Such a realization correlates well with our understanding of molecular evolution, which often obliterates easily recognizable sequence similarity among genes that diverged a long time ago, but leaves behind traces of statistically significant patterns of conserved residues that are apparent only when multiple, related sequences are aligned. To reflect the concept of different degrees of divergence between genes, proteins are often subjected to multilevel classification, with the term “family” reserved for groups of proteins related by short evolutionary distances that still retain traces of similarity in their primary sequences. But families can be organized into groups of higher hierarchy that are linked by more far reaching relationships. For instance, in PFAM [29] such groups are called “clans,” whereas “superfamily” is often used in other resources. We can expect that further development of even more sensitive algorithms for recognition of distant homologs would expand the list of clans or equivalent groupings in other classification systems. The growth of the protein universe can then be investigated on the level of individual proteins, protein families, or clans/superfamilies, and we can expect qualitatively different answers on each level.

“Hypothetical proteins” can also be grouped into families, and the latest release of PFAM contains 2,156 families annotated as domains of unknown function (DUF), with 91 further families listed as Uncharacterized Protein Families (n.b. since 95% of families of unknown function in PFAM are called DUFs, from here on we will use the term “DUF” to denote both DUF and Uncharacterized Protein Families). Classifying DUF families into superfamilies and clans is more problematic, as such classification often depends on additional information, such as three-dimensional structures and/or protein function, and such information is not obviously available.

Structural genomics, represented in the United States by the NIH NIGMS PSI (http://www.nigms.nih.gov/Initiatives/PSI), has pioneered a novel approach to structural biology that is highly complementary to strategies pursued in individual structural biology labs. Instead of focusing on individual proteins, US structural genomics and, specifically, the four large-scale production centers of the PSI have focused their attention on substantially increasing structural coverage of protein space. DUF families have then become natural targets as such families cover a significant fraction of the unexplored protein universe. In contrast, “classical” structural biology efforts are mainly focused on well-characterized systems, leaving the majority of protein families outside of their sphere of interest, including, by default, almost all DUF families.

Here, we investigate structures of representatives of DUF families determined by the PSI as a means to gain insights into the yet unexplored regions of protein space. While not perhaps as statistically rigorous a sampling as will eventually be possible, the substantial size of the sample (∼250 protein families) offers a rare opportunity to make some general observations and conclusions and enables predictions to be made about the trends and features of the uncharted regions of the protein universe. In particular, we are now able to determine the distribution of the folds in these families and deduce the evolutionary relationships of many DUF families to previously characterized families. For many of these families, determination of their three-dimensional structures offers the first hypotheses about their function and represents a powerful approach to initiate and promote studies for experimental verification of the biological function of these unexplored and underappreciated regions of the protein universe.

## Results

### PSI and Novel Protein Families

As of October 2008, the PSI centers have determined structures of over 250 protein families designated by PFAM as families of unknown function (DUF or UPF). In addition, the PSI centers have solved the first structures of about 100 protein families that consist solely of “hypothetical proteins,” which have not yet been included in PFAM, although they fulfill all of the definitions of a DUF protein family. These families are now being systematically added to new releases of the PFAM database (see statistics and discussion about DUF families at http://xfam.wordpress.com/). The PSI centers have also solved structural representatives of over 300 protein families that have only limited functional information. However, for clarity and for ease of comparison to PFAM and Structural Classification of Proteins (SCOP) annotations, we have focused our analyses here on the group of 248 protein families identified and classified by PFAM as DUFs and already classified by SCOP for their structural novelty.

**Figure 1:**
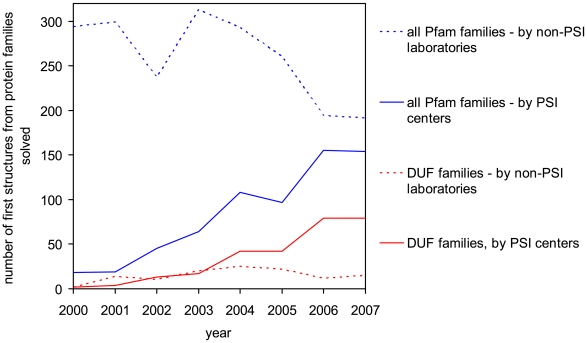
The number of DUF structures solved by PSI centers (continuous red line) and by other laboratories (dashed red line). For comparison, the contribution of the PSI centers to structural determination of PFAM protein families is shown as a continuous blue line and by other laboratories as a dashed blue line.

**Figure 2:**
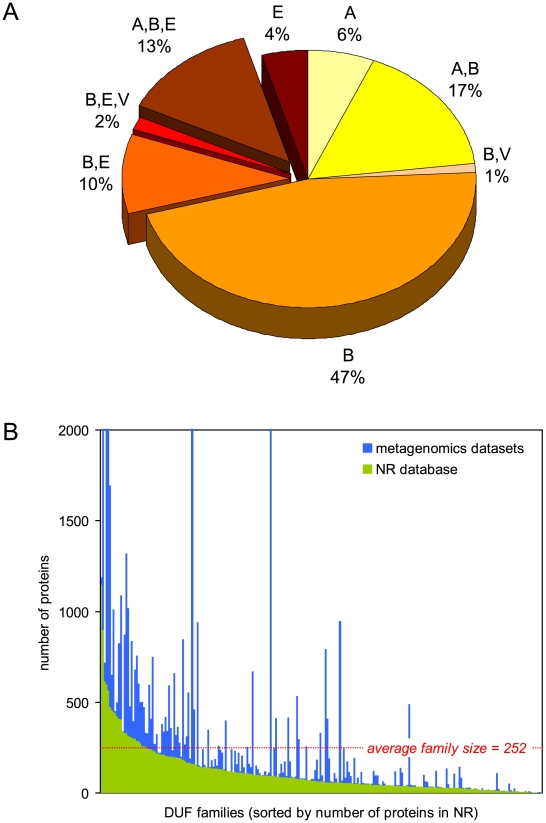
Distribution and sizes of DUF families. (A) Distribution of DUF families in the kingdoms of life. An “A” denotes families present in Archaea, “B” denotes Bacteria, “E” Eukaryota, and “V” Viruses. “B,E” denotes families present in both Bacteria and in Eukaryota and so forth. (B) Distribution of sizes of DUF families according to the PFAM database. Green bars show number of family members found in the NR database (without metagenomic sequences), and blue bars indicate additional members found in metagenomic datasets.

**Figure 3:**
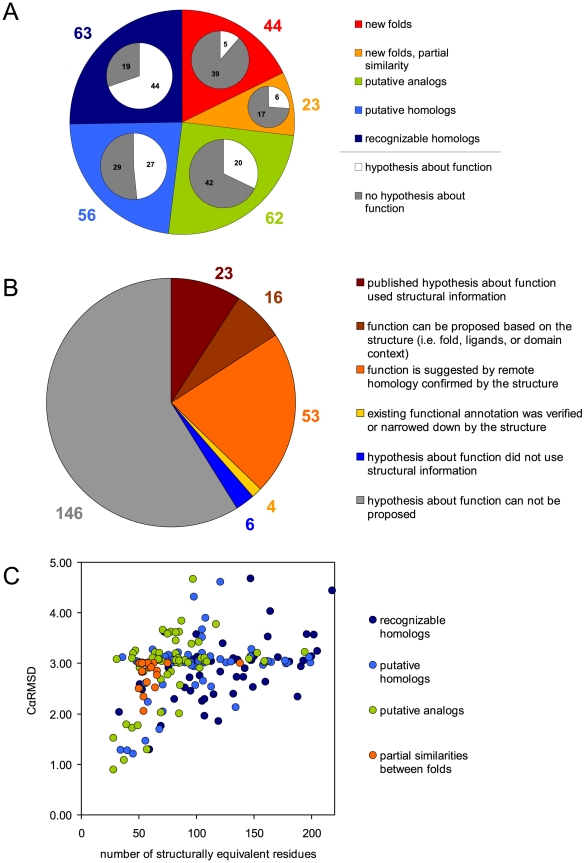
Structural and functional characterization of DUF families. (A) Distribution of DUF structures with regard to structural similarity and homology to previously known structures. The main pie chart shows overall percentages of DUF families with new folds, new folds partially similar to previously known folds, putative analogs, putative homologs, and recognizable homologs. The inset pie charts show the percentage of DUF families with proposed hypothesis about function in each of these six categories. (B) Impact of solved structures on hypotheses about function proposed for DUF families. (C) Distribution of C_α_ RMSD versus number of equivalent residues in structural alignments between first structural representatives of DUF families and the closest previously solved structures of the same fold. Dark blue circles indicate pairs with detectable sequence homology (recognizable homologs). Pairs with marginal homology confirmed by the solved structure (putative homologs) are shown by bright blue circles. Pairs with unresolved homology are shown as green circles. As expected, structural alignments of pairs with detectable homology tend to be longer and C_α_ RMSD values tend to be lower. For illustration, we also show the same data for 20 partial similarities between new folds found in DUF structures and previously known folds (orange circles). We note that, by definition, the set of partial similarities is limited to pairs with more than 50 equivalent residues and C_α_ RMSD below 3 Å.

The PSI centers are the major contributor of the first three-dimensional structures for families of unknown function (Figure 1). The four PSI production centers have provided first structural information for more than 600 of the protein families classified in the PFAM database [20], including more than 300 in the last 2 y alone. A total of 248 of these protein families were annotated as DUFs. Together these families contain proteins from all kingdoms of life, and 43% are represented in more than one kingdom (Figure 2A). These families have a wide distribution of sizes with an average of around 252 members (Figure 2B). On average, 112 members from each DUF family come from the non-redundant protein database of NCBI (http://www.ncbi.nlm.nih.gov/) with an additional 140 members from recently published metagenomics datasets. The DUF families show substantial variation in size (Figure 2B). In particular, the addition of metagenomics data significantly increases the size of some families. The relatively large size and wide distribution of these DUF families in all kingdoms of life indicates that, despite their present lack of characterization, they constitute important components of the molecular machinery of life. Furthermore, it is now apparent that some of the DUF families exhibit some level of similarity to other protein families, as evaluated by sequence and structural criteria (Figure 3A and 3B).

### Divergence versus Novelty

Each of the 248 DUF structures solved by PSI was analyzed by structure comparison and remote homology recognition tools (see Materials and Methods). In contrast to their designation, 25% of these DUF families can be linked to other protein families using a sensitive, profile-profile alignment algorithm [30] or other similar tools at a significance level where we expect <5% false predictions. In all cases, structure similarity confirmed earlier fold and function assignment system (FFAS) predictions, thus validating and even exceeding the significance thresholds established earlier on historical benchmarks of fold recognition [27]. The next 48% of DUF families, despite lack of statistically significant sequence similarity to any previously characterized family in FFAS search, could still be recognized as having known folds by combination of automated structure comparisons and manual structure analysis. As we show later, for about half of them, one can still find evidence of remote homology to previously solved proteins of the same fold that was overlooked in initial analyses because it fell below the significance threshold, or because the homology can only be identified via nontrivial indirect links (see Materials and Methods).

**Figure 4:**
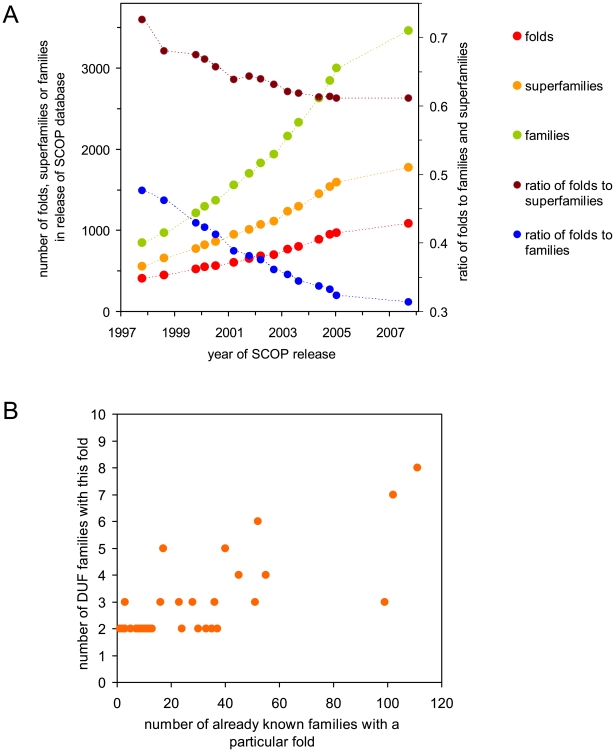
Analysis of trends in families, superfamilies, and DUFs. (A) Long-term trends in the proportion of protein folds to protein families and to protein superfamilies according to SCOP database. Each point corresponds to one release SCOP database (n.b., there were no SCOP releases between January 2005 and September 2007). This analysis is based on the data available from the SCOP website (http://scop.mrc-lmb.cam.ac.uk/scop/). (B) Number of fold representatives in DUF families as a function of a number of already known families with the same fold (n.b., the number of known families of the same fold was derived from the SCOP database).

The remaining 27% of the 248 DUF families represent novel folds. The assignment of new folds was carried out internally at the Joint Center for Structural Genomics (JCSG) and has been confirmed by the recently released, pre-SCOP classification [31]. These observations agree with the trend indicated by the analysis of SCOP database where the proportion of the number of folds to the number of protein families and superfamilies has been decreasing substantially over the past 10 y (Figure 4A). Full results of sequence and structure similarity analysis of DUF families are available in Supporting Information (Table S1).

### New “Old” Folds

As previously indicated, 181 of the DUF families in this study, i.e., 73%, adopt structures that are similar to previously determined protein structures, although the majority of these similarities could not be predicted in advance by standard distant homology recognition tools. Previously known folds that were found at least twice among DUF families are presented in Table 1 (n.b., the complete list of fold assignments for DUF families is available in Supporting Information—see Table S1). This distribution was compared to the frequency of the same folds in the SCOP database (see Figure 4B). It is interesting to note that some of the most popular folds, such as P-loop hydrolases or ribonuclease H, are not present among the 248 DUF families. Other popular folds, such as TIM barrels or NADP binding domains, are present at very low frequency, whereas some folds, such as the flavodoxin fold, are represented several times more often than expected.

**Table 1:** Representation of known protein folds in 248 PSI structures from DUF families.

Fold	Number of Representatives in 248 DUFs	Number of Previously Known Protein Families with This Fold in SCOP Database
Ferredoxin-like	8	111
DNA/RNA-binding 3-helical bundle	7	102
TIM αβ-barrel	3	99
Immunoglobulin-like β-sandwich	4	55
S-adenosyl-L-methionine-dependent methyltransferase	6	52
Alpha/alpha superhelix	3	51
Flavodoxin-like	4	45
Double-stranded β-helix	5	40
SH3-like barrel	2	37
Four-helical up-and-down bundle	3	36
α/β hydrolase	2	35
Restriction endonuclease-like	2	33
OB-fold	2	30
Rubredoxin-like	3	28
Long alpha hairpin	2	24
Beta-Grasp (ubiquitin-like)	2	24
Thioredoxin fold	3	23
TBP-like	5	17
Cysteine proteinases	3	16
Spectrin repeat-like	2	16
Immunoglobulin/albumin-binding domain-like	2	13
Bromodomain-like	2	12
NAD(P)-binding Rossmann-fold domains	2	12
Nucleotidyltransferase	2	11
Bacillus chorismate mutase-like	2	10
Ferritin-like	2	10
Anticodon-binding domain-like	2	9
DSRBD-like	2	8
Fwde/GAPDH domain-like	2	8
Lipocalins	2	8
5-bladed β-propeller	2	7
α/β knot	2	5
Dodecin subunit-like	2	5
T-fold	2	5
Secretion chaperone-like	3	3
Cyclophilin-like	2	3
DSREFH-like	2	2
MK0786-like	2	1
Hcp1-like	2	1
VPA0735-like	2	1
YheA-like	2	1

The table contains only folds that are represented at least twice in different DUF families. The folds here cover 114 DUF families (67 known folds were represented by only one DUF structure each and 67 families have novel folds).

In some instances, our ability to more readily recognize certain conserved features or patterns in highly divergent protein sequences may account for the apparent skewing of this distribution from values expected for a random, nonbiased set of proteins and protein families. For instance, protein folds that are strongly associated with a single function and are characterized by a well-defined sequence motif, such as the Walker box of P-loop ATPases [32] or the HCXAGXGR(S/T)G sequence in protein tyrosine phosphatases [33], are easy to recognize with sequence-based methods, and families displaying such patterns are rarely left unannotated, and would not be assigned to a DUF group. On the other hand, folds that contain proteins that do not share a common sequence motif and/or folds that can be divided into a large number of functionally distinct superfamilies (n.b., these two groups mostly coincide) are also more likely to be represented in DUF families (see Figure 4B).

The most interesting question related to those DUF structures, which we have now shown adopt a previously known fold, is whether they diverged from already known families and, therefore, can be classified into already known clans or superfamilies, or whether they are examples of convergent evolution [34]–[36]. While rigorous proof of homology is often difficult, if not impossible, usually the combination of several arguments enables us to arrive at a satisfactory answer. Using this approach, we can propose that, for most of the cases investigated here, the similarities of these DUF families to known folds and families represent actual, albeit often very distant, homologies.

As indicated, 25.4% (63) of these DUF families can now be linked to other families using newer, more sensitive comparison methods. In such analyses, only sequence information is taken into account and the similarity measured between patterns of substitutions at specific positions along the sequence is identified. Similarities in structures often reaffirm sequence-based arguments for homology. In fact, an additional 21% (51) of the families can be linked to members of known folds by marginal sequence similarity that is verified by the observed structural similarity. Another 2% (five structures) of the DUFs were solved with cofactors/ligands that closely match those found in proteins of the same fold, thus suggesting similar function and, hence, a good chance of having some evolutionary relationship. Thus, only 25% (62) of the DUF structures are classified as known folds and, at the same time, cannot be connected to previously known proteins of the same fold with current day, state-of-the-art, remote homology recognition methods and should, therefore, be considered at present as putative analogs of known structures.

To check whether any systematic differences exist between the sets of proteins that exhibit (i) both sequence and structure similarities (denoted as “recognizable homologs”), (ii) structural similarity but only marginal sequence similarity (denoted as “putative homologs”), and (iii) structural similarity but no currently identifiable sequence similarity (denoted as “putative analogs”), we compared the distribution of structural alignment lengths and root mean square deviation of protein Cα atoms (C_α_ RMSDs) for these three groups with known representatives of their category (see Figure 3C). In agreement with trends described in a recent paper [36], the number of corresponding residues in the structural alignments between DUF structures assigned as putative analogs with their potential homologs tend to be smaller and the corresponding C_α_ RMSD values of these pairs tend to be higher than the values for the other two categories of proteins assigned as putative homologs and recognizable homologs (see Figure 3C). However, the profiles of structural similarities in these three groups are very similar, suggesting that most proteins from the group of putative analogs may be, in fact, distant, but not readily recognizable, homologs of previously characterized protein families. At the same time, it is clear that some proteins in this group, especially those that consist of a small number of secondary structure elements, such as α-helical hairpins, probably arise from convergent evolution.

### Old “New” Folds

Sixty seven (27%) out of the 248 DUFs analyzed here can be classified as having a new fold. This classification is based on structural comparisons (see Materials and Methods). These assignments were then confirmed by pre-SCOP classifications when they became available [31]. The surprisingly small, although certainly not insignificant, percentage of new folds among the DUF families is a major result of this study. It is also interesting to note that this modest percentage of new folds among DUF families has been steadily decreasing over time—over 50% of the DUFs analyzed in this study were solved in the last 2 y, but they contain only 20% of the new folds found in the 248 DUFs. We would certainly still expect to find new folds as we continue to explore protein sequence space, as our knowledge of the protein structure universe is far from complete. All estimates of the number of possible protein folds [37]–[40] point to numbers much greater than the ∼1,350 folds that we know today (pre-SCOP resource 11/17/08). Despite the decreasing percentage of new folds being discovered in DUF families, these families still represent one, if not the most, rich and diverse set of targets for attempting to uncover most of the remaining folds in the protein universe.

**Figure 5:**
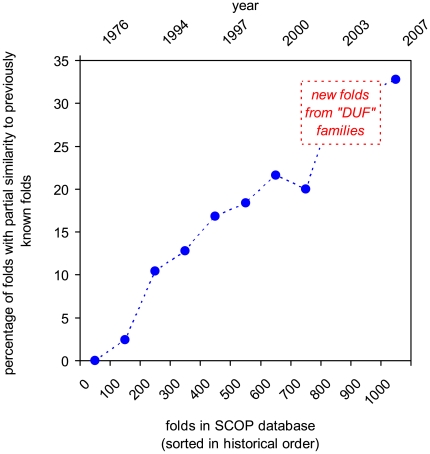
Evidence of saturation of protein fold space as a function of time. With growing number of folds, the percentage of folds with partial structural similarity to other folds is increasing, and hence, the number of truly new folds being discovered is rapidly decreasing. Folds were added in historical order in groups of 100 and the percentage of folds with partial similarity to any previously solved fold was calculated for each group. All cases in which FATCAT algorithm found at least 50 equivalent residues superimposed with C_α_ RMSD <3 Å were regarded as putative cases of “significant partial similarity” and were subject to visual verification. As indicated by a box on the graph, 30% of new folds from DUF families described here show such partial similarities to other protein folds.

**Figure 6:**
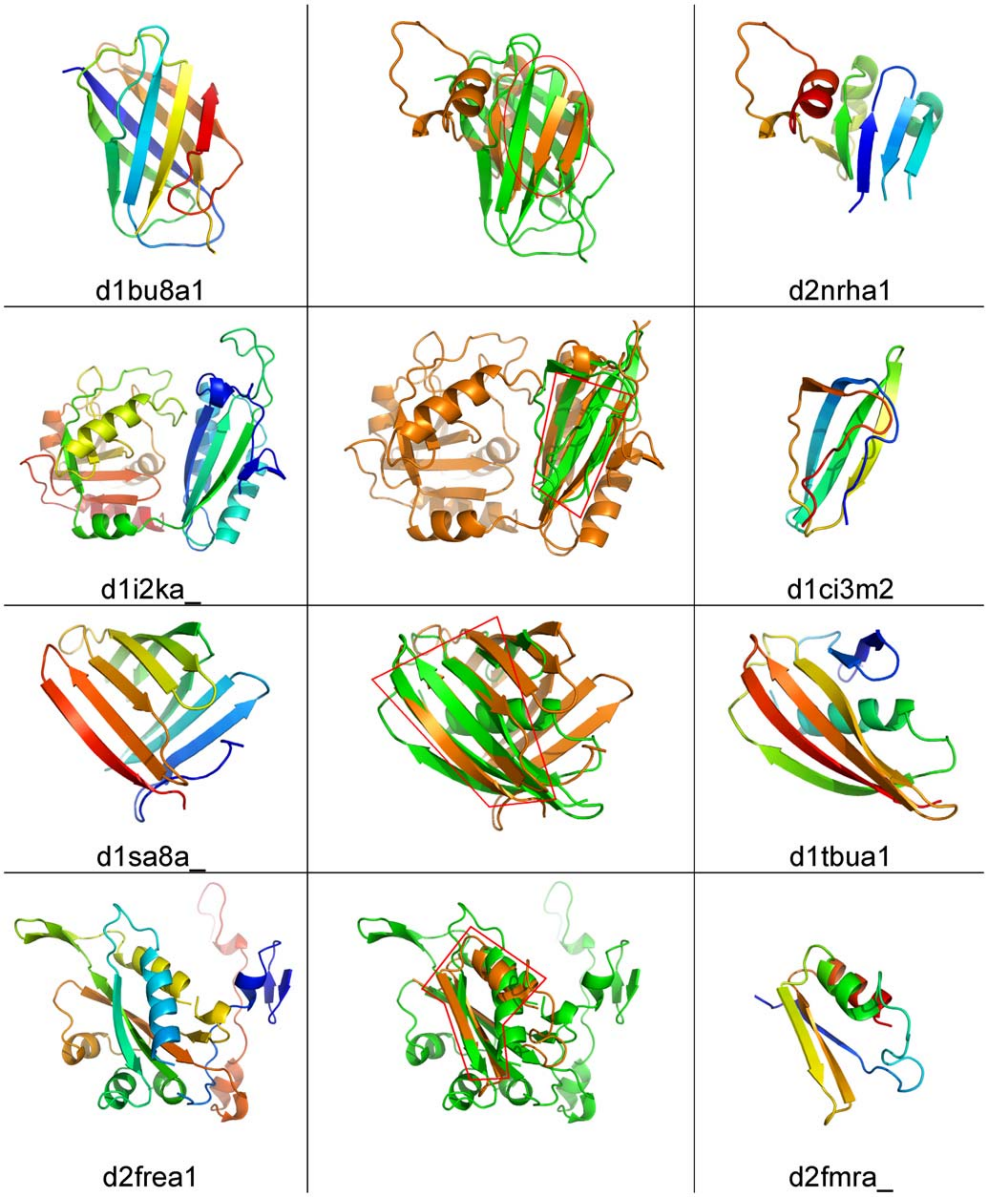
Examples of structural similarities detected in sub-domains of different folds, as classified by the SCOP database. The leftmost column shows the first structure from each pair of partially similar structures, and the rightmost column shows the second structure from each pair. The central column contains structural superposition of each pair. A region of structurally equivalent residues identified by FATCAT is indicated by an red contoured box .

However, the automated structure comparisons performed with flexible structure alignment by chaining aligned fragment pairs allowing twists (FATCAT) [41] reveal yet another interesting finding. Over a third of these new folds contain fragments with significant structural similarity to fragments of known proteins that adopt different overall folds. The presence of some structural similarity among different folds is well known, and most authors suggest that, in most cases, it has its origin in the general evolution of protein structure [35],[42]–[47]. In all of the DUF examples that we have analyzed, the similarities were statistically significant, but fall short of representing a full fold match. With more than 50 equivalent residues superimposed with C_α_ RMSDs below 3 Å, most of these sub-fold similarities extend beyond the obvious, well-defined supersecondary structure motifs. Upon closer examination, we found that this finding not only is true for new folds from DUF families but also holds for many recently solved proteins that were identified to have new folds. As illustrated in Figure 5, only 15% of the new folds identified in 1995 showed such partial similarity to previously known folds, but this percentage has grown to almost 30% in the new folds solved in the last 2 y. It is important to note that these sub-fold similarities still have a discrete distribution, so this result does not necessarily argue for (or necessarily disprove) a continuum in protein fold space, in an analogous way that the presence of common factors between two integer numbers does not infer the space of integer numbers is continuous. Contrary to the examples described in the previous section, in none of the cases in this group could we find evidence of extremely distant homology to known proteins. Thus, the most likely explanation of this phenomenon is that, with an increasing number of known protein structures, we are observing a saturation of the available fold space that is first seen at the level of micro-domains that represent shorter, usually compact, structures that become component pieces of different folds. Therefore, we would contend that most similarities in this group are examples of “constrained” evolution, where similar solutions are found independently because of the limited number of choices available. Some selected examples of such sub-fold similarities are illustrated in Figure 6.

### Structure and Hypotheses about Function in DUF Families

So far, we have focused entirely on the structures of these newly solved DUF domains and have not addressed the question of biological function. It is difficult to rigorously address this problem at present, as prediction of function of most of these families from their sequence and structure has not yet been verified by experiment. Nevertheless, other approaches to protein function prediction, such as genome or genomic neighborhood context analysis, strongly suggest that the overall biochemical function is well conserved between pairs of proteins with significant structural similarity. Thus, hypotheses about function can often be proposed based on the analysis of the protein structures themselves. Protein function prediction is a rapidly changing field [48]–[50], and hence, for simplicity of interpretation, we decided not to use complex function prediction algorithms for the DUF families but, instead, collected functional predictions from the existing literature and, if such predictions were not available, we proposed hypotheses about biological functions of DUF families based only on homology and structural similarity.

Most of the structural representatives of DUF families adopt previously known protein folds and, as we argued above, in the majority of cases represent distant homologs of already characterized protein families. Since even very remote homology usually translates into similarity in at least some aspects of function, identifying such relationships provides a basis to formulate hypotheses about the biological function of a family. Moreover, sequence similarity often links a DUF family to a specific protein superfamily, enabling the functional hypotheses to be more precise. As 25% (63) of DUF families can be linked by remote homology to other proteins from the same fold (“recognizable homologs”) and, for another 23% (56), some evidence of remote homology could be found (“putative homologs”), these associations are good starting points for hypotheses about function. For seven “recognizable homologs” and eight “putative homologs,” a hypothesis about function was proposed in the original publication describing the structure. Our own analyses indicate some functional hints for a further 37 of the “recognizable homologs” and 19 of the “putative homologs.” Thus, 71 out of 119 of these DUF families now have at least a hypothesis about a putative function (Figure 3A and 3B) as a result of their structures being determined.

In the group of “putative analogs”—the 62 DUF families with known folds, but no other detectable homology to other proteins—hypotheses about function can presently be based only on the structural information. In the first approximation, the possibility of inferring function from the protein fold depends on the numbers and functional diversity of proteins that have already been observed to adopt this fold. Some protein folds, although highly populated, are strongly associated with a specific function or functional category, while other folds contain proteins of diverse functions. This division is usually apparent in annotation resources for protein families. In particular, folds from the first group are usually contained in only one superfamily in the SCOP database and are also often grouped into one clan (a group of related protein families) in the PFAM database. In contrast, folds from the second group are usually distributed over several superfamilies in the SCOP database and are represented in several PFAM families that are not grouped into a single clan. We can find representatives of both groups among DUF structures. For example, the third most abundant fold in the 248 DUF structures analyzed here is the S-adenosyl-L-methionine-dependent methyltransferase fold that contains only one functional superfamily of the same name and corresponds to a single large PFAM clan of methyltransferases. In this case, based on simple extrapolation, DUF families that adopt this fold are most likely to be methyltransferases, where the key challenge is to predict their precise mechanism and substrate specificity. The second most popular fold among DUF families, the DNA/RNA-binding 3-helical bundle, is represented in multiple superfamilies, but still strongly suggests a specific activity, namely, nucleic acid recognition. Other examples of folds found in DUF families and associated with specific functions are shown in Table 2. In contrast, other folds, such as the ferredoxin-like fold or TIM beta/alpha-barrel, contain so many functionally diverse families that structural similarity alone does not readily translate into a functional hypothesis, although it may still restrict the repertoire of possible functions. As a result, in this group of DUF families, we could propose hypotheses solely based on their folds for only 15 of the 62 families, and for another five families, we found functional annotations in the original publications describing the solved structure. Thus, even for this most challenging set (putative analogs), around a third (20 of 62) of the DUFs now have some hypothesis about function.

**Table 2:** SCOP folds associated with a single functional category or limited functional categories that were used to propose hypotheses about the functions of DUF families.

SCOP Fold	Dominant Functional Category or Categories
DNA/RNA-binding 3-helical bundle	Usually bind nucleic acids
S-adenosyl-L-methionine-dependent methyltransferase	Usually methyltransferases and methylases
Cysteine proteinases	Enzymes; usually peptidases, esterases
5-bladed β-propeller	Enzymes; usually sugar binding or nucleotide hydrolyzing
α/β knot	Methyltransferases
DSREFH-like	Involved in sulfur reduction or oxidation
Fwde/GAPDH domain-like	Enzymes; dehydrogenases, deaminases
Lipocalins	Bind hydrophobic ligands in their interior
NAD(P)-binding Rossmann-fold domains	Bind NAD(P)
Nucleotidyltransferase	Nucleic acid processing enzymes
Restriction endonuclease-like	Nucleic acid processing enzymes
T-fold	Enzymes
Ribosomal protein L5	Ribosomal proteins, may bind nucleic acids
Prokaryotic lipoproteins and lipoprotein localization factors	Lipoproteins and lipoprotein carrier proteins
Bacterial protein-export protein SecB	Involved in protein export
PurS-like	Involved in purine metabolism
FAD-linked reductases, C-terminal domain	Oxidoreductases
DNA-binding domain	Bind DNA
Mota C-terminal domain-like	Bind DNA, transcription factors
ssDNA-binding transcriptional regulator domain	Transcriptional regulators
Double-split β-barrel	Bind DNA
DNA-glycosylase	Bind DNA

Interestingly, some functional assignments were possible even in the group of DUF families with novel folds. We found that, for nine out of these 67 DUF families with novel folds, some hypotheses about function were proposed in the literature or in public databases and, for two others, we can make an hypothesis about function based on a bound ligand in the structure, or from the fold of a second domain present in the same protein that contains the DUF domain.

In summary, the existing literature contains functional hypotheses only for about 10% of DUF families. However, by combining structural similarity with other lines of reasoning, we can propose functional hypothesis for an additional 31%. Thus, some hypotheses about function can be established for 102 (41%) of the 248 DUF families.

Experimental determination of a protein structure plays a crucial role in establishing a hypothesis about its function (see Figure 3B). In fact, almost all (96 out of 102) hypotheses were, at least partly, based on the structural information. Among 29 published functional hypotheses, 23 used structural information. Published hypotheses about function that did not use structural information were based on direct experiments (four families) and on genome context (one family). In addition, for one DUF family, functional assignment became available before the structure was determined. For families for which we could not find any hypothesis about function in existing literature, we checked whether it was possible to suggest a functional category based on structural similarity and homology. Our analysis showed that structural information alone (e.g., fold, ligands, and domain context) provided hints about function for another 16 DUF families. For yet another 53 families, structures aided in verifying remote homology and enabled a DUF family to be linked to a functional category. For a further four families, existing functional annotation was verified or narrowed by the structure. Taken together, we now have reasonable hypotheses for almost half of the DUF families analyzed in this study.

For more than half (146) of all DUF families, we cannot yet propose reliable hypotheses about function, mostly because many of these families have folds that are functionally diverse and, thus, the fold itself does not provide sufficient functional information. The remaining DUF families represent completely novel folds and will need experimental function determination or more sophisticated computational tools.

## Discussion

The PSI in the last few years has embarked on an unprecedented exploration of uncharacterized regions of protein space. Structures determined by the PSI include first representatives of 248 DUFs, as classified by PFAM, as well as hundreds of first representatives of other protein families, many of which have unknown function and contain solely “hypothetical proteins” but for various reasons were not classified as DUFs. In this study, we focused entirely on the families designated as DUF by PFAM as they present a well-defined set of novel protein families. Analysis of these families, now possible because experimentally determined structures of family representatives are available, shows that, despite their designation, an overwhelming majority of them exhibit significant structural similarity to already known protein structures. In combination with other types of analysis, we hypothesize that a majority of these families represent highly divergent homologs of previously characterized protein families. This surprising finding implies that most of the presently uncharacterized regions of the protein universe are composed of distant homologs of known protein families, while only a relatively small fraction would represent truly novel families. This conclusion underscores the importance of developing more sensitive tools for recognizing distant homologies and predicting the functional consequences of such relations. From 248 families that appeared novel using specific tools used by PFAM in 2007, 63 can be linked to known protein families using a more sensitive tool and an additional 118 using only structural analysis. While this relative distribution may change with the development of better algorithms, it is important to stress that whether the protein families analyzed here are called DUFs or are given specific names, these are genuine, novel families. Our focus on DUF families simply used a specific stage in the development of our knowledge of protein families to focus on a large, consistently defined group of novel protein families.

The results presented here also indicate that remote homology, especially if confirmed by solved structures, is the most promising method of proposing hypotheses about functions of DUF families (see Figure 3A and 3B). Remote homology prediction, if available, often links DUF family not only to a specific fold, but also to a particular protein family that may have a precise functional assignment. Structural similarity validates (or invalidates) distant homology predictions simply by verifying whether it had linked the DUF family to the correct (or incorrect) fold. As a result, homology predictions validated by solved structures make hypotheses about function more reliable and more precise.

Our statistics can be also used to revisit the age old question as to the number of protein folds, which have been estimated by several different approaches to be between 1,000 [51] and 10,000 [37]–[40]. The PSI has determined the first structures for more than 600 protein families that were selected by novelty of their sequences and lack of any structural information. These efforts have made it abundantly clear that the vast majority of structurally uncharacterized protein space consists of families that will be eventually classified into already known folds. The most recent results from the PSI suggest that this trend is accelerating and the percentage of new folds in structures representing new families solved in 2008 is substantially lower than the historical trend. From 1,741 DUF families listed in the 22.0 release of PFAM, 248 were analyzed as a part of this study. From the remaining 1,493 families, 474 can be linked to known folds by the profile-profile remote homology detection program FFAS [30]. Among the remaining 1,019 DUF families, 192 are predicted to have transmembrane domains (>2 predicted transmembrane helices in more than 50% of representative domain sequences) and 93 are intrinsically disordered (>50% of predicted structural disorder in more than 50% of representative domain sequences) – with some families having both, leaving 761 DUF families that may potentially have new folds. But if the trend established in last 7 y continues, all of the remaining DUF families would provide only about 200 novel folds, which would increase the number of folds in the Protein Data Bank (PDB) by only ∼20%, but still substantially exceed the current trend of depositions of new folds into the PDB. Such a systematic approach would provide a very rapid mechanism to complete the repertoire of protein folds by focusing on the relatively small subset of DUF sequences and families not yet structurally characterized.

In addition to the trend described above, analyses of genuinely novel structures that represent new folds show that a significant and growing percentage contains complex structural elements or substructures that are present in other folds. Both observations clearly suggest that the repertoire of known protein folds is reaching a plateau and that an infinite continuum of topologies or structures probably does not exist, although that issue is being hotly debated [45],[46]. The idea that geometrical constraints on possible modes of packing of secondary structure elements limit the space of existing protein folds was discussed as early as 1987 by Finkelstein and Ptitsyn [52]. As suggested in the Results section, it is quite likely that known folds of small all-alpha proteins are getting close to exhausting all geometrically feasible packing modes. Such, purely geometrical, saturation may then be a plausible explanation for some structural similarities between short proteins consisting of a small number of secondary structure elements [53]. On the other hand, structural similarities between seemingly unrelated proteins encompassing a large number of β strands and α helices arranged in a complex topology may yet be suggestive of a very remote evolutionary relationship, even if these two proteins are classified as different folds and homology cannot be detected even with the most sensitive sequence-based methods.

While not a formal proof, our results strongly suggest that protein structure space is saturating much faster than previously predicted. Therefore, instead of identifying completely “new territories” of protein structural space, the role of high throughput structural biology has now become that of linking those “uncharted territories” of protein sequence space to previously characterized regions. Our results suggest that, despite the rapidly growing sequence databases, structural coverage of protein space may be entering the period where structural and functional diversity within known protein folds and reshuffling of well-defined substructures are emerging as the central focus of structural and molecular biology.

## Materials and Methods

### Family Assignment and Statistics of DUF Families

Sequences of constructs of all solved protein structures, their deposition times, and the identity of the depositing laboratories were downloaded from the PDB FTP site (ftp://ftp.wwpdb.org). Multiple sequence alignments and HMM representing PFAM families, as well as HMMER suite, were downloaded from the PFAM website (http://pfam.janelia.org/, http://hmmer.janelia.org/).

The sequences of PDB structures were then clustered at the 90% sequence identity level using the CD-HIT program [54], and the earliest deposited structure was selected from each cluster. All representatives were then searched against HMM of PFAM families using hmmpfam program from the HMMER suite. All hits with e-values lower than 0.05 were further analyzed. If more than one HMM was aligned to a particular region of PDB sequence, then this region was assigned to a family represented by the HMM that gave a smaller e-value. The first structural representative of each PFAM family was identified by comparing deposition times of all PDB entries assigned to that family. PFAM families with first structures solved by the PSI centers were then identified. Based on these data, we calculated yearly contributions of PSI centers and other laboratories in determining first structures from PFAM families (Figure 1). The list of DUF and UPF families solved by PSI centers was then assembled and information about sizes of DUF families and their presence in different kingdoms of life were extracted directly from downloaded alignments of PFAM families (ftp://ftp.sanger.ac.uk/pub/databases/Pfam/current_release/Pfam-A.full.gz).

### Structure Comparison and Fold Classification of DUF Structures

Structural representatives of 248 DUF families were compared to all structural domains from SCOP database version 1.73 using FATCAT algorithm [41] without allowing flexibility in the alignments.

More than half (135 out of 248) of the structures from the DUF families analyzed here had been already annotated in SCOP database (version 1.73) or pre-SCOP resource (current till December 2008) and were indicated by trivial hits found by FATCAT. We treated SCOP classification as a “golden standard” and adopted SCOP fold assignments when they were available. If the first structural representative of a given DUF family had not been classified in the SCOP database, all structural similarities detected by FATCAT were examined and fold assignments were proposed based on alignment length, Cα RMSD, and visual inspection. If the structure of a protein from a DUF family was the first representative of a given fold in SCOP database, then the DUF family was classified as having a new fold. If the structure of a DUF family was found to be similar to a previously determined structure, then it was classified as a known fold. DALI searches [55] were run for the remaining DUF families for which FATCAT did not find a convincing similarity to any SCOP fold, and if fold assignment could not be made, then these structures were tentatively classified as new folds.

### Remote Homology Detection

All DUF families with known folds were checked for homology linking them to previously known representatives of that fold. FFAS profiles were calculated for all DUF families and compared with libraries of FFAS profiles representing PDB, PFAM, COG, and SCOP databases. In addition, PFAM HMMs representing DUF families were also compared to HMMs of all PFAM families using hhpred program version 1.5 [28] (http://toolkit.tuebingen.mpg.de/hhpred).

If FFAS aligned a profile of a DUF family with any profile representing PDB structure or SCOP domain with a score better (i.e., lower) than −9.5 and this structure had been deposited before the first structural representative of DUF family, then this DUF family was classified as a homolog of previously known fold (in our study, such families are denoted “recognizable homologs”). In all such cases, remote homology prediction was confirmed by structural similarity recognized by FATCAT and DALI algorithms.

For the remaining DUF families, we inspected 10 top scoring FFAS profiles from the PDB and SCOP databases even if their scores were worse than the significance threshold of −9.5. If any of these hits was found to be of the same fold as the structure of DUF family, then the family was classified as a putative homolog of a known fold (i.e., the structure was required to confirm marginal homology prediction). We also performed intermediate remote homology searches by checking whether the DUF family can be linked to the representative of the same fold by finding an FFAS profile of any PFAM or COG family that is similar both to DUF family and to any previously solved structure of the same fold. In a similar fashion, we checked whether any DUF family can be linked to earlier representatives of the same fold via intermediate homologs found with hhpred program [28].

All DUF families that could be linked to proper folds by marginal homology confirmed by the structure or by intermediate homology searches were denoted as “putative homologs.”

The remaining DUF families with known folds, but with no evidence of homology linking them to other representatives of the same fold, were denoted as “putative analogs” to indicate that the question about homology between them and other members of the same fold remains open.

### Detection of Partial Structural Similarities between Different Protein Folds

In order to address the issue of partial similarities among different protein folds, we sorted folds from the SCOP database (version 1.73) in an historical order according to deposition dates of their earliest representatives. Then, for each fold, we identified the shortest domain and used it as a representative of this fold in all following calculations. In the first step, a representative of each fold was compared to representatives of previously determined folds using FATCAT algorithm (without allowing alignment flexibility). Structural alignments for which FATCAT found at least 50 equivalent residues superimposed with C_α_ RMSD below 3 Å were regarded as candidates for significant partial similarities. They were then subjected to visual inspection, which revealed that 80% of FATCAT alignments fulfilling the above criteria correspond to real, nontrivial, partial similarities (i.e., at least three consecutive secondary structure elements were superimposed and included in the set of equivalent residues). The remaining 20% were mostly alignments covering a single long helix or a helical hairpin. Protein folds for which we found at least one structural match of more than 50 residues with C_α_ RMSD below 3 Å were classified as “partially similar to previously solved folds.” Representatives of SCOP folds (still sorted in historical order) were then grouped into bins of 100 and the percentages of folds with partial similarity to earlier folds were calculated for each bin (Figure 5) taking into account the fact that 20% of them are expected to be trivial similarities.

In a similar way, we searched for partial structural similarities between 67 DUF domains classified as novel folds and representatives of previously determined SCOP folds. Structural alignments of more than 50 residues with C_α_ RMSD lower than 3 Å were identified and subject to visual verification. Thirty percent of DUF structures (a total of 20) that were classified as new folds were found to be partially similar to known structures with different overall folds, consistent with a long-term trend observed in the SCOP database (Figure 5).

### Establishing Hypotheses about Function of DUF Families

In order to establish hypotheses about functions of DUF families, we searched existing literature for functional annotations, and for proteins that lacked a primary publication, we established hypotheses about function based on remote homology confirmed by structural similarity or, if it was not possible, based solely on the structural similarity. Standard FFAS profiles were prepared for all DUF families by performing five iterations of PSI-BLAST on the NR database clustered at 85% sequence identity using CD-HIT [54]. First, we used FFAS to compare the profile of a DUF family with libraries of profiles of annotated protein families and structures such as PFAM, COG, and PDB. If a significant similarity to functionally annotated protein or protein family was found, then its function was proposed as the most likely hypothesis about the function of the DUF family. For DUF families without significant homology to functionally annotated families, we examined cases when marginal homology was confirmed by the solved structure (as described in the previous section). If we found evidence of homology to a functionally annotated family, then its function was proposed as the most likely function of the DUF family. Finally, for families of known folds, but without any evidence of homology to functionally annotated families, we checked if high conservation of function of annotated structures of the same fold allowed for extrapolation. If a given fold contained more than three protein families grouped into one functional superfamily in the SCOP database, or all functionally annotated PFAM families from that fold were grouped into one functional PFAM clan linked to a single functional category, then we assumed that the DUF family adopting this fold is likely to have a function similar to the function associated with that fold.

Independently from the above procedure, we examined ligands found in structures from DUF families and checked whether they pointed at a specific function of some functionally annotated family of the same fold.

The procedure described above was not applicable to DUF families of novel fold, but still, for 11 of them, hypotheses about function were found in the existing literature.

## Supporting Information

Table S1 **A full list of DUF families with first structural representatives solved by the PSI analyzed in this study.**
(0.10 MB PDF)

## Abbreviations

Cα RMSD: root mean square deviation of protein Cα atoms
DUF: domain of unknown function (Pfam database designation for families without functional annotation)
FATCAT: flexible structure alignment by chaining aligned fragment pairs allowing twists
FFAS: fold and function assignment system
JCSG: Joint Center for Structural Genomics
ORF: open reading frame
PDB: Protein Data Bank
PSI: Protein Structure Initiative
SCOP: Structural Classification of Proteins
