# Supplementary material for: Exploration of Uncharted Regions of the Protein Universe
Source: PLoS Biol. 2009 Sep 29;7(9):e1000205. doi: 10.1371/journal.pbio.1000205 (PMC2744874; doi:10.1371/journal.pbio.1000205)
Supplement: Table S1 — A full list of DUF families with first structural representatives solved by the PSI analyzed in this study. (0.10 MB PDF) [file pbio.1000205.s001.pdf]

| Family id | Family description (from Pfam database) | Family is represented in these kingdoms of life (A-Archaea, B-Bacteria, E-Eukaryota, V-Viruses) | Number of family members in NR (according to Pfam) | PDB id of the first structure | Fold assignment (SCOP 1.73 nomenclature) | Relationship to previously known structures (0=new fold; 1=partially similar fold; 2=putative analog; 3=putative homolog; 4=homolog) | Is it possible to propose a hypothesis about function? (1-yes, 0-no) | Hypothesis about function (quotes from publications indicated by quotation marks) | Basis of the hypothesis about function |
|---|---|---|---|---|---|---|---|---|---|
| PF01519 | Protein of unknown function DUF16 | A,B | 42 | 2ba2 | single helix | 4 | yes | "strong possibility that the coiled-coil structure of the DUF 16 domain of MPN010 is involved in motility of M. pneumoniae" | sequence and structure analysis / publication linked to PDB entry |
| PF01796 | Domain of unknown function DUF35 | A,B | 1010 | 2gnr | OB-fold | 4 | yes | predicted to bind nucleic acids | remote homology confirmed by the structure |
| PF01861 | Protein of unknown function DUF43 | A,B | 30 | 2qm3 | S-adenosyl-L-methionine-dependent methyltransferase | 4 | yes | probably involved in transfer of methyl group | remote homology confirmed by the structure |
| PF01865 | Protein of unknown function DUF47 | A,B | 595 | 2iiu | Spectrin repeat-like | 4 | yes | FE coordinates PO3 on protein surface; PhoU-like phosphate regulatory protein | remote homology confirmed by the structure, ligands |
| PF01877 | Protein of unknown function DUF54 | A | 175 | 2nrq | Ribosomal protein L5 | 2 | yes | possibly ribosomal protein, may bind nucleic acids | fold |
| PF01883 | Domain of unknown function DUF59 | A,B,E | 3219 | 1uwd | Alpha-lytic protease prodomain-like | 3 | yes | "predicted metal-sulfur cluster biosynthetic enzyme, predicted to be involved in ring hydroxylating complexes." | genome context / publication linked to PDB entry |
| PF01893 | Uncharacterized protein family UPF0058 | A | 41 | 2gf4 | Immunoglobulin/albumin-binding domain-like | 2 | no | | |
| PF01904 | Protein of unknown function DUF72 | A,B,E | 478 | 1vpq | TIM beta/alpha-barrel | 3 | no | | |
| PF01906 | Domain of unknown function DUF74 | A,B,E | 756 | 1y2i | Dodecin subunit-like | 2 | no | | |
| PF01908 | Protein of unknown function DUF75 | A,B,E | 848 | 2p90 | Phosphorylase/hydrolase-like | 2 | no | | |
| PF01910 | Domain of unknown function DUF77 | A,B,E | 326 | 1lxj | Ferredoxin-like | 2 | yes | "may have a role in yeast cell-wall biogenesis." | experiment/ publication linked to PDB entry |
| PF01931 | Protein of unknown function DUF84 | A,B,E | 186 | 1u14 | Anticodon-binding domain-like | 3 | no | | |
| PF01933 | Uncharacterized protein family UPF0052 | A,B,E | 1089 | 2ffe | Cofd-like | 0 | yes | "2-phospho-(S)-lactate transferase" | function was known before / publication linked to PDB entry |
| PF01934 | Protein of unknown function DUF86 | A,B | 409 | 1ylm | Four-helical up-and-down bundle | 4 | no | | |
| PF01936 | Protein of unknown function DUF88 | A,B | 678 | 2qip | PIN domain-like | 3 | yes | alignment contains many conserved aspartates, suggesting an enzymatic function such as an endonuclease or glycosyl hydrolase, structural similarity points at exonuclease | existing annotations (Pfam) narrowed by structural similarity |
| PF01937 | Protein of unknown function DUF89 | A,B,E | 270 | 1xfi | AF1104-like | 0 | no | unknown,charged side chains surround UNL | unknown / publication linked to PDB entry |
| PF01947 | Protein of unknown function DUF98 | A,B,E | 37 | 2nwi | Chorismate lyase-like | 4 | yes | possibly a subfamily of chorismate lyases | remote homology confirmed by the structure |
| PF01949 | Protein of unknown function DUF99 | A,B | 52 | 2qh9 | Ribonuclease H-like motif | 3 | no | | |
| PF01951 | Protein of unknown function DUF101 | B | 222 | 1jw3 | MTH1598-like | 0 | yes | RNA-binding | sequence and structure analysis / publication linked to PDB entry |
| PF01954 | Protein of unknown function DUF104 | A,B | 45 | 2nwt | Domain of alpha and beta subunits of F1 ATP synthase-like | 2 | no | | |
| PF01958 | Domain of unknown function DUF108 | A,B,E | 223 | 1j5p | Fwde/GAPDH domain-like | 3 | yes | putative oxidoreductase or aspartate dehydrogenase | fold and evidence of homology, ligands |
| PF01980 | Uncharacterized protein family UPF0066 | A,B,E | 397 | 1xqb | Yaeb-like | 0 | no | | |
| PF01982 | Domain of unknown function DUF120 | A,B | 122 | 2oyn | Reductase/isomerase/elongation factor common domain | 3 | no | | |

| Family id | Family description (from Pfam database) | Family is represented in these kingdoms of life (A-Archaea, B-Bacteria, E-Eukaryota, V-Viruses) | Number of family members in NR (according to Pfam) | PDB id of the first structure | Fold assignment (SCOP 1.73 nomenclature) | Relationship to previously known structures (0=new fold; 1=partially similar fold; 2=putative analog; 3=putative homolog; 4=homolog) | Is it possible to propose a hypothesis about function? (1-yes, 0-no) | Hypothesis about function (quotes from publications indicated by quotation marks) | Basis of the hypothesis about function |
|---|---|---|---|---|---|---|---|---|---|
| PF01983 | Protein of unknown function DUF121 | A,B | 247 | 2i5e | Nucleotide-diphospho-sugar transferases | 4 | yes | probably nucleotide-diphospho-sugar transferase | remote homology confirmed by the structure |
| PF01987 | Protein of unknown function DUF124 | A,B,E | 498 | 1pg6 | Double-stranded beta-helix | 3 | yes | binds nucleic acids, possibly RNA | existing annotations enhanced by structural similarity |
| PF01989 | Protein of unknown function DUF126 | A,B,E | 257 | 2hi6 | The "swivelling" beta/beta/alpha domain | 4 | yes | phosphotransferase | remote homology confirmed by the structure |
| PF01994 | Protein of unknown function DUF127 | A | 119 | 2o3a | Alpha/beta knot | 4 | yes | putative methyltransferase | remote homology confirmed by the structure |
| PF01995 | Domain of unknown function DUF128 | A,B | 48 | 2qyx | Ferredoxin-like | 2 | no | | |
| PF02130 | Uncharacterized protein family UPF0054 | B,E | 2877 | 1oz9 | Zincin-like | 4 | yes | "similar to collagenases and gelatinases, may have a similar function but performs it under different conditions." | sequence and structure analysis / publication linked to PDB entry |
| PF02410 | Domain of unknown function DUF143 | A,B,E | 2591 | 2id1 | Nucleotidyltransferase | 2 | yes | binds nucleic acids, possibly RNA polymerase, role in development | structural similarity and existing literature |
| PF02457 | Domain of unknown function DUF147 | A,B | 873 | 2fb5 | Yojj-like | 1 | yes | the distribution of conserved histidines and aspartates suggests that this may be a metal dependent phosphoesterase. YacK from Bacillus subtilis also contains a Helix-hairpin-helix HHH motif that is characteristic of DNA binding proteins. | existing annotations confirmed by structural similarity |
| PF03479 | Domain of unknown function (DUF296) | A,B,E | 651 | 2h6l | AF0104/ALDC/Ptd012-like | 3 | yes | may bind RNA | fold and evidence of homology |
| PF03629 | Domain of unknown function (DUF303) | B,E,V | 661 | 1zmb | Flavodoxin-like | 3 | no | | |
| PF03658 | Uncharacterized protein family (UPF0125) | B | 357 | 2hj1 | Beta-Grasp (ubiquitin-like) | 4 | no | | |
| PF03671 | Uncharacterized protein family (UPF0185) | E | 65 | 1l7y | Beta-Grasp (ubiquitin-like) | 3 | yes | "may interact with an unidentified activating enzyme in a new ubiquitin-like modification system" | sequence and structure analysis / publication linked to PDB entry |
| PF03685 | Uncharacterized protein family (UPF0147) | A,B | 100 | 2qsb | Bromodomain-like | 2 | no | | |
| PF03713 | Domain of unknown function (DUF305) | B,V | 1019 | 2qf9 | Ferritin-like | 3 | yes | putative ferritin secreted by bacteria | existing annotations enhanced by fold and evidence of homology |
| PF03884 | Domain of unknown function (DUF329) | B | 419 | 1lv3 | Glucocorticoid receptor-like (DNA-binding domain) | 3 | yes | "involved in transcription either through protein-protein interactions (e.g., NF) or by DNA-binding" | sequence and structure analysis / publication linked to PDB entry |
| PF03885 | Protein of unknown function (DUF327) | B | 62 | 2p61 | Four-helical up-and-down bundle | 2 | no | | |
| PF03886 | Protein of unknown function (DUF330) | B | 331 | 2iqi | Anticodon-binding domain-like | 3 | no | | |
| PF03928 | Domain of unknown function (DUF336) | A,B,E | 826 | 2a2l | Profilin-like | 3 | yes | homologous to sensor proteins | remote homology confirmed by the structure |
| PF03937 | Protein of unknown function (DUF339) | B,E | 1321 | 1puz | Ygfy-like | 1 | yes | "may possibly function to mediate protein-protein interactions in a manner that is distinct from that of the classical TPR motif" | sequence and structure analysis / publication linked to PDB entry |
| PF04010 | Protein of unknown function (DUF357) | A | 116 | 2oo2 | Immunoglobulin/albumin-binding domain-like | 2 | no | | |

| Family id | Family description (from Pfam database) | Family is represented in these kingdoms of life (A-Archaea, B-Bacteria, E-Eukaryota, V-Viruses) | Number of family members in NR (according to Pfam) | PDB id of the first structure | Fold assignment (SCOP 1.73 nomenclature) | Relationship to previously known structures (0=new fold; 1=partially similar fold; 2=putative analog; 3=putative homolog; 4=homolog) | Is it possible to propose a hypothesis about function? (1-yes, 0-no) | Hypothesis about function (quotes from publications indicated by quotation marks) | Basis of the hypothesis about function |
|---|---|---|---|---|---|---|---|---|---|
| PF04013 | Protein of unknown function (DUF358) | A,B | 116 | 2qmm | Alpha/beta knot | 4 | yes | probably methyltransferase | remote homology confirmed by the structure, ligand |
| PF04016 | Domain of unknown function (DUF364) | A,B | 68 | 2h1q | NAD(P)-binding Rossmann-fold | 3 | no | | |
| PF04036 | Domain of unknown function (DUF372) | A | 27 | 2i52 | MK0786-like | 0 | no | | |
| PF04038 | Domain of unknown function (DUF381) | A | 29 | 2i52 | MK0786-like | 0 | no | | |
| PF04040 | Domain of unknown function (DUF375) | A,B | 1695 | 2o34 | T-fold | 2 | no | | |
| PF04041 | Domain of unknown function (DUF377) | A,B,E | 161 | 1vkd | 5-bladed beta-propeller | 4 | yes | probably involved in sugar binding or nucleotide hydrolysis | remote homology confirmed by the structure |
| PF04126 | Domain of unknown function (DUF369) | A,B | 108 | 1zx8 | Cyclophilin-like | 3 | yes | "it will be of interest to investigate whether TM1367 has rotamase activity, binds CsA, or acts as a regulator of calcineurin" | sequence and structure analysis / publication linked to PDB entry |
| PF04167 | Protein of unknown function (DUF402) | A,B | 221 | 2p12 | FomD barrel-like | 0 | no | | |
| PF04222 | Protein of unknown function, DUF | B | 107 | 2q9r | N-terminal domain of adenylylcyclase associated protein, CAP | 2 | no | | |
| PF04229 | Uncharacterized protein family (UPF0157) | A,B,E | 235 | 2nrk | Nucleotidyltransferase | 4 | yes | probably involved in processing of nucleic acids | remote homology confirmed by the structure |
| PF04237 | Protein of unknown function (DUF419) | B | 266 | 2a1v | Secretion chaperone-like | 2 | no | | |
| PF04242 | Protein of unknown function (DUF424) | A | 49 | 2qya | IF3-like | 2 | no | | |
| PF04255 | Protein of unknown function (DUF433) | A,B | 249 | 2ga1 | DNA/RNA-binding 3-helical bundle | 3 | yes | probably binds nucleic acids | fold and evidence of homology |
| PF04273 | Putative phosphatase (DUF442) | B | 398 | 2f46 | Phosphotyrosine protein - phosphatases II | 4 | yes | putative protein phosphatase | remote homology confirmed by the structure |
| PF04287 | Domain of unknown function, DUF446 | B | 142 | 2hgk | Bromodomain-like | 2 | no | | |
| PF04289 | Protein of unknown function (DUF447) | A,B | 96 | 2iml | Split barrel-like | 3 | yes | probably a flavoprotein | fold and evidence of homology, ligands |
| PF04296 | Protein of unknown function (DUF448) | B | 602 | 1g2r | Ylxr-like | 0 | yes | "it is proposed that YlxR is an RNA-binding protein" | sequence and structure analysis / publication linked to PDB entry |
| PF04303 | Protein of unknown function (DUF453) | B,E | 593 | 2h9f | Diaminopimelate epimerase-like | 4 | no | | |
| PF04327 | Protein of unknown function (DUF464) | B,V | 145 | 1s12 | Eif1-like | 2 | no | | |
| PF04337 | Protein of unknown function, DUF480 | B | 152 | 2nr3 | DNA/RNA-binding 3-helical bundle | 3 | yes | probably binds nucleic acids | fold and evidence of homology |
| PF04356 | Protein of unknown function (DUF489) | B | 240 | 1qz4 | Ycfc-like | 0 | no | | |
| PF04359 | Protein of unknown function (DUF493) | B,E | 339 | 1rwu | Ferredoxin-like | 3 | yes | "plays a role in the allosteric regulation of lipoic acid biosynthesis or the glycine cleavage system" | sequence and structure analysis / publication linked to PDB entry |

| Family id | Family description (from Pfam database) | Family is represented in these kingdoms of life (A-Archaea, B-Bacteria, E-Eukaryota, V-Viruses) | Number of family members in NR (according to Pfam) | PDB id of the first structure | Fold assignment (SCOP 1.73 nomenclature) | Relationship to previously known structures (0=new fold; 1=partially similar fold; 2=putative analog; 3=putative homolog; 4=homolog) | Is it possible to propose a hypothesis about function? (1-yes, 0-no) | Hypothesis about function (quotes from publications indicated by quotation marks) | Basis of the hypothesis about function |
|---|---|---|---|---|---|---|---|---|---|
| PF04378 | Protein of unknown function (DUF519) | B | 379 | 2oo3 | S-adenosyl-L-methionine-dependent methyltransferase | 4 | yes | probably involved in transfer of methyl group | remote homology confirmed by the structure |
| PF04379 | Protein of unknown function (DUF525) | B,E | 837 | 1tza | Immunoglobulin-like beta-sandwich | 3 | no | | |
| PF04398 | Protein of unknown function, DUF538 | E | 341 | 1ydu | At5g01610-like | 0 | no | | |
| PF04416 | Protein of unknown function (DUF509) | A | 35 | 1zd0 | PF0523-like | 1 | no | | |
| PF04430 | Protein of unknown function (DUF498/DUF598) | A,B,E | 474 | 1ihn | MTH938-like | 0 | yes | "could potentially bind double-stranded nucleic acid" | sequence and structure analysis / publication linked to PDB entry |
| PF04445 | Protein of unknown function (DUF548) | B,E | 460 | 2oyr | S-adenosyl-L-methionine-dependent methyltransferase | 4 | yes | probably involved in transfer of methyl group | remote homology confirmed by the structure, ligand |
| PF04524 | Protein of unknown function, DUF586 | B | 717 | 2p5z | Phage tail proteins | 3 | yes | putative phage assembly protein | remote homology confirmed by structural similarity |
| PF04525 | Protein of unknown function (DUF567) | A,B,E | 222 | 1zxu | Tubby C-terminal domain-like | 4 | yes | probably involved in distribution of phospholipids, related to scramblases | remote homology confirmed by the structure |
| PF04591 | Protein of unknown function, DUF596 | B | 30 | 2o5h | DNA/RNA-binding 3-helical bundle | 2 | yes | probably binds nucleic acids | fold |
| PF04634 | Protein of unknown function, DUF600 | B | 116 | 2ia1 | MHC antigen-recognition domain | 2 | no | | |
| PF04672 | Protein of unknown function (DUF574) | B | 142 | 2qe6 | S-adenosyl-L-methionine-dependent methyltransferase | 4 | yes | probably involved in transfer of methyl group | remote homology confirmed by the structure, ligand |
| PF04751 | Protein of unknown function (DUF615) | B | 342 | 2p0t | PSPTO4464-like | 0 | no | | |
| PF04919 | Protein of unknown function, DUF655 | A,B | 96 | 2i5h | OB-fold, SAM domain-like fold | 4 | no | | |
| PF05082 | Protein of unknown function (DUF683) | B | 53 | 2js5 | ROP-like | 4 | yes | probably involved in signaling or transcription regulation | remote homology confirmed by the structure |
| PF05167 | Uncharacterized ACR (DUF711) | A,B | 132 | 2ha9 | PFL-like glycyl radical enzymes | 2 | no | | |
| PF05256 | Uncharacterized protein family (UPF0223) | B | 91 | 2oy9 | BH2638-like | 1 | no | | |
| PF05303 | Protein of unknown function (DUF727) | E | 35 | 1sgo | N domain of copper amine oxidase-like | 2 | no | | |
| PF05430 | Protein of unknown function (DUF752) | A,B | 750 | 2qy6 | S-adenosyl-L-methionine-dependent methyltransferases | 4 | yes | probably involved in transfer of methyl group | remote homology confirmed by the structure |
| PF05618 | Protein of unknown function (DUF785) | A,B | 554 | 2pma | Acid proteases | 4 | yes | protease | remote homology confirmed by the structure |
| PF05638 | Protein of unknown function (DUF796) | B | 499 | 1y12 | Hcp1-like | 1 | yes | "probably part of protein secretion apparatus encoded by virulence locus in P.aeruginosa Hcp1 associates with proteins on both faces in order to build a channel through which other macromolecules could be transported" | experiment / publication linked to PDB entry |

| Family id | Family description (from Pfam database) | Family is represented in these kingdoms of life (A-Archaea, B-Bacteria, E-Eukaryota, V-Viruses) | Number of family members in NR (according to Pfam) | PDB id of the first structure | Fold assignment (SCOP 1.73 nomenclature) | Relationship to previously known structures (0=new fold; 1=partially similar fold; 2=putative analog; 3=putative homolog; 4=homolog) | Is it possible to propose a hypothesis about function? (1-yes, 0-no) | Hypothesis about function (quotes from publications indicated by quotation marks) | Basis of the hypothesis about function |
|---|---|---|---|---|---|---|---|---|---|
| PF05838 | Predicted lysozyme (DUF847) | B,E,V | 296 | 2ikb | Lysozyme-like | 4 | yes | probably a lyzosyme | remote homology confirmed by the structure |
| PF05891 | Eukaryotic protein of unknown function (DUF858) | E | 120 | 1xtp | S-adenosyl-L-methionine-dependent methyltransferase | 4 | yes | probably involved in transfer of methyl group | remote homology confirmed by the structure, ligand |
| PF05899 | Protein of unknown function (DUF861) | B,E | 1190 | 1lkn | Double-stranded beta-helix | 4 | no | | |
| PF05907 | Eukaryotic protein of unknown function (DUF866) | E | 99 | 1zso | MAL13P1.257-like | 0 | no | | |
| PF05913 | Bacterial protein of unknown function (DUF871) | A,B | 131 | 1x7f | Two domains: TIM beta/alpha-barrel and Cyclophilin-like | 3 | no | | |
| PF05962 | Bacterial protein of unknown function (DUF886) | B,E | 179 | 1yll | Double-stranded beta-helix, duplication | 3 | no | | |
| PF05979 | Bacterial protein of unknown function (DUF896) | B | 146 | 2hep | Long alpha hairpin | 2 | no | | |
| PF06004 | Bacterial protein of unknown function (DUF903) | B | 151 | 2jn0 | Sm-like fold | 2 | no | | |
| PF06006 | Bacterial protein of unknown function (DUF905) | B | 50 | 2hjj | DNA-binding domain | 2 | yes | probably binds nucleic acids | fold |
| PF06014 | Bacterial protein of unknown function (DUF910) | B | 98 | 2nn4 | Spectrin repeat-like | 2 | no | | |
| PF06032 | Protein of unknown function (DUF917) | A,B,E | 175 | 2o3i | CV3147-like | 0 | no | | |
| PF06041 | Bacterial protein of unknown function (DUF924) | B,E | 941 | 2i6h | Alpha/alpha superhelix | 2 | no | | |
| PF06078 | Bacterial protein of unknown function (DUF937) | B | 114 | 1z67 | Yidb-like | 1 | yes | "may be a nucleic acid binding protein" | sequence and structure analysis / publication linked to PDB entry |
| PF06108 | Protein of unknown function (DUF952) | A,B,E | 671 | 2o0p | ADP-ribosylation | 3 | no | | |
| PF06133 | Protein of unknown function (DUF964) | A,B | 109 | 2iaz | YheA-like | 0 | no | | |
| PF06201 | Domain of Unknown Function (DUF1000) | E | 200 | 1xoy | Galactose-binding domain-like | 4 | yes | probably involved in "sugar binding or protein-protein interactions" | sequence and structure analysis / publication linked to PDB entry |
| PF06228 | Protein of unknown function (DUF1008) | B | 77 | 2hqv | Heme iron utilization protein-like, duplication | 4 | yes | "binding site for heme, while conserved in HemS and ChuS, is not conserved in AGR_C_4470p, suggesting that it probably has a different function" | sequence and structure analysis / publication linked to PDB entry |
| PF06267 | Family of unknown function (DUF1028) | A,B | 409 | 2imh | Ntn hydrolase-like | 3 | no | probably an enzyme | fold and evidence of homology |
| PF06283 | Protein of unknown function (DUF1037) | A,B | 112 | 1t0b | Flavodoxin-like | 4 | no | | |
| PF06304 | Protein of unknown function (DUF1048) | B | 29 | 2hh6 | Glutamyl tRNA-reductase dimerization domain | 2 | no | | |

| Family id | Family description (from Pfam database) | Family is represented in these kingdoms of life (A-Archaea, B-Bacteria, E-Eukaryota, V-Viruses) | Number of family members in NR (according to Pfam) | PDB id of the first structure | Fold assignment (SCOP 1.73 nomenclature) | Relationship to previously known structures (0=new fold; 1=partially similar fold; 2=putative analog; 3=putative homolog; 4=homolog) | Is it possible to propose a hypothesis about function? (1-yes, 0-no) | Hypothesis about function (quotes from publications indicated by quotation marks) | Basis of the hypothesis about function |
|---|---|---|---|---|---|---|---|---|---|
| PF06335 | Protein of unknown function (DUF1054) | B | 55 | 2a8e | Yktb/PF0168-like | 2 | yes | some structural similarity and marginal homology to proteins from secretion chaperone-like fold, possibly involved in secretion | remote homology confirmed by the structure |
| PF06352 | Protein of unknown function (DUF1061) | B | 54 | 1t06 | Alpha/alpha superhelix | 4 | no | | |
| PF06475 | Protein of unknown function (DUF1089) | B | 77 | 2h1t | Prokaryotic lipoproteins and lipoprotein localization factors | 2 | yes | probably related to lipoproteins | fold |
| PF06520 | Protein of unknown function (DUF1105) | A,B,E | 92 | 2if6 | Cysteine proteinases | 4 | yes | probably involved in cleavage of peptide or ester bond | remote homology confirmed by the structure |
| PF06526 | Protein of unknown function (DUF1107) | B | 81 | 2jro | TBP-like | 2 | no | | |
| PF06557 | Protein of unknown function (DUF1122) | A,B | 13 | 2arh | Acyl-coa N-acyltransferases (Nat) | 3 | no | | |
| PF06572 | Protein of unknown function (DUF1131) | B | 41 | 2qzb | beta-lactamase-inhibitor protein, BLIP | 2 | no | | |
| PF06619 | Protein of unknown function (DUF1149) | B | 54 | 2hng | Bacterial protein-export protein secb | 2 | yes | may be involved in protein export | fold |
| PF06627 | Protein of unknown function (DUF1153) | B | 66 | 2oa4 | DNA/RNA-binding 3-helical bundle | 4 | yes | DNA-binding | remote homology confirmed by the structure |
| PF06684 | Protein of unknown function (DUF1185) | B | 947 | 2qtp | Bacillus chorismate mutase-like | 2 | no | | |
| PF06742 | Protein of unknown function (DUF1214) | A,B,E | 363 | 2p3y | VPA0735-like | 0 | no | | |
| PF06748 | Protein of unknown function (DUF1217) | B | 82 | 2o8s | AGR_C_984p-like | 0 | no | | |
| PF06764 | Protein of unknown function (DUF1223) | B,E | 127 | 2axo | Two domains: thioredoxin fold and immunoglobulin-like beta-sandwich | 4 | yes | possible thioltransferase | remote homology confirmed by the structure |
| PF06794 | Uncharacterized protein family (UPF0270) | B | 141 | 1y0n | Yehu-like | 0 | no | | |
| PF06821 | Alpha/Beta hydrolase family of unknown function (DUF1234) | B,E | 330 | 1uxo | Alpha/beta hydrolase | 4 | yes | "it is very likely that YdeN is a carboxylesterase active on a water-soluble ester in which the substrate, possibly the acyl group, has a hydrophobic nature" | sequence and structure analysis / publication linked to PDB entry |
| PF06824 | Protein of unknown function (DUF1237) | B,E | 162 | 2nvp | Alpha/alpha toroid | 4 | no | | |
| PF06844 | Protein of unknown function (DUF1244) | B | 793 | 2o35 | SMc04008-like | 0 | no | | |
| PF06849 | Protein of unknown function (DUF1246) | A,B | 175 | 2pbz | preATP-grasp domain | 3 | yes | ligase; IMP biosynthesis protein PURP; protein is trimer with ATP in each monomer. | fold and evidence of homology, ligand |
| PF06855 | Protein of unknown function (DUF1250) | B | 159 | 2fj6 | SAM domain-like | 2 | no | | |
| PF06863 | Protein of unknown function (DUF1254) | A,B,E | 313 | 2p3y | VPA0735-like | 0 | no | | |

| Family id | Family description (from Pfam database) | Family is represented in these kingdoms of life (A-Archaea, B-Bacteria, E-Eukaryota, V-Viruses) | Number of family members in NR (according to Pfam) | PDB id of the first structure | Fold assignment (SCOP 1.73 nomenclature) | Relationship to previously known structures (0=new fold; 1=partially similar fold; 2=putative analog; 3=putative homolog; 4=homolog) | Is it possible to propose a hypothesis about function? (1-yes, 0-no) | Hypothesis about function (quotes from publications indicated by quotation marks) | Basis of the hypothesis about function |
|---|---|---|---|---|---|---|---|---|---|
| PF06865 | Protein of unknown function (DUF1255) | B,E | 260 | 2oyz | Double-stranded beta-helix | 4 | no | | |
| PF06877 | Protein of unknown function (DUF1260) | B | 191 | 1nxi | Ferredoxin-like | 2 | yes | "could be involved in amino acid metabolic pathways such as that for arginine" | sequence and structure analysis / publication linked to PDB entry |
| PF06908 | Protein of unknown function (DUF1273) | B | 101 | 2nx2 | Moco carrier protein-like | 4 | yes | possible decarboxylales | remote homology confirmed by the structure |
| PF06924 | Protein of unknown function (DUF1281) | B | 32 | 2ijr | Api92-like | 0 | no | | |
| PF06938 | Protein of unknown function (DUF1285) | B | 534 | 2ra9 | new fold; some similarity to PH domain-like barrel | 1 | no | | |
| PF06948 | Protein of unknown function (DUF1291) | A,B | 26 | 2fb6 | Dsrefh-like | 4 | yes | may be involved in sulfate binding and may play a role in sulfur oxidation | remote homology confirmed by the structure |
| PF06973 | Protein of unknown function (DUF1297) | A | 190 | 2pbz | ATP-grasp domain | 3 | yes | ATP-utilizing enzymes involved in purine biosythesis | fold and evidence of homology |
| PF06998 | Protein of unknown function (DUF1307) | B | 70 | 2joe | Secretion chaperone-like | 2 | no | | |
| PF07005 | Protein of unknown function, DUF1537 | B | 318 | 1yzy | Ygbk-like | 1 | no | | |
| PF07031 | Protein of unknown function (DUF1321) | B | 333 | 2nys | Stringent starvation protein B, sspb | 4 | yes | possibly starvation protein | remote homology confirmed by the structure |
| PF07045 | Protein of unknown function (DUF1330) | A,B,E | 2550 | 2fiu | Ferredoxin-like | 4 | no | | |
| PF07049 | Protein of unknown function (DUF1332) | B | 2043 | 2h5n | EF-hand, EF Hand-like fold | 4 | yes | tellurite resistance protein | remote homology confirmed by the structure |
| PF07050 | Protein of unknown function (DUF1333) | B | 94 | 2pih | YheA-like | 1 | no | | |
| PF07063 | Protein of unknown function (DUF1338) | B,E | 93 | 2rjb | Glyoxalase/Bleomycin resistance protein/Dihydroxybiphenyl dioxygenase | 2 | no | | |
| PF07072 | Protein of unknown function (DUF1342) | B | 233 | 2oez | YacF-like | 1 | no | | |
| PF07080 | Protein of unknown function (DUF1348) | B,E | 157 | 2imj | Cystatin-like | 4 | no | | |
| PF07090 | Protein of unknown function (DUF1355) | B | 185 | 2gk3 | Flavodoxin-like | 4 | no | | |
| PF07100 | Protein of unknown function (DUF1362) | B | 64 | 1nc7 | Hypothetical protein TM1070; similar to immunoglobulin-like beta-sandwich | 0 | no | | |
| PF07166 | Protein of unknown function (DUF1398) | B | 29 | 2hh8 | YdfO-like | 0 | no | | |
| PF07191 | Protein of unknown function (DUF1407) | B | 51 | 2jne | Rubredoxin-like | 4 | no | | |
| PF07208 | Protein of unknown function (DUF1414) | B | 90 | 2ota | YejL-like | 0 | no | | |

| Family id | Family description (from Pfam database) | Family is represented in these kingdoms of life (A-Archaea, B-Bacteria, E-Eukaryota, V-Viruses) | Number of family members in NR (according to Pfam) | PDB id of the first structure | Fold assignment (SCOP 1.73 nomenclature) | Relationship to previously known structures (0=new fold; 1=partially similar fold; 2=putative analog; 3=putative homolog; 4=homolog) | Is it possible to propose a hypothesis about function? (1-yes, 0-no) | Hypothesis about function (quotes from publications indicated by quotation marks) | Basis of the hypothesis about function |
|---|---|---|---|---|---|---|---|---|---|
| PF07237 | Protein of unknown function (DUF1428) | A,B | 97 | 2okq | Ferredoxin-like | 3 | no | | |
| PF07262 | Protein of unknown function (DUF1436) | B | 15 | 2gkp | NMB0488-like | 0 | no | | |
| PF07286 | Protein of unknown function (DUF1445) | B,E | 347 | 2pif | PSTPO5379-like | 0 | no | | |
| PF07313 | Protein of unknown function (DUF1460) | B | 109 | 2im9 | Cysteine proteinases | 4 | yes | probably involved in cleavage of peptide or ester bond | remote homology confirmed by the structure |
| PF07315 | Protein of unknown function (DUF1462) | B | 44 | 1xg8 | Thioredoxin fold | 4 | no | | |
| PF07336 | Protein of unknown function (DUF1470) | B | 152 | 2pw4 | Jann2411-like | 0 | no | | |
| PF07338 | Protein of unknown function (DUF1471) | B | 452 | 2noc | Dodecin subunit-like | 2 | no | | |
| PF07350 | Protein of unknown function (DUF1479) | B,E | 413 | 2csg | Double-stranded beta-helix | 4 | yes | putative oxidoreductase; Succinic acid coordinating with FE in the active site | remote homology confirmed by the structure, ligands |
| PF07369 | Protein of unknown function (DUF1488) | B | 154 | 2gpi | Dsrbd-like | 2 | no | | |
| PF07408 | Protein of unknown function (DUF1507) | B | 47 | 2gbo | Open three-helical up-and-down bundle | 2 | no | | |
| PF07515 | Protein of unknown function (DUF1528) | B | 89 | 2ipq | DNA/RNA-binding 3-helical bundle | 2 | yes | probably binds nucleic acids | fold |
| PF07566 | Domain of Unknown Function (DUF1543) | B | 193 | 2qsd | Purs-like | 2 | yes | probably involved in purine metabolism | fold |
| PF07755 | Protein of unknown function (DUF1611) | A,B | 491 | 2g0t | P-loop containing nucleoside triphosphate hydrolases | 4 | yes | p-loop containing nucleoside triphosphate hydrolase | remote homology confirmed by the structure |
| PF07892 | Protein of unknown function (DUF1667) | A,B,E | 45 | 2jov | T-fold | 2 | no | | |
| PF07955 | Protein of unknown function (DUF1687) | E | 29 | 1wpi | Thioredoxin fold | 3 | yes | "redox protein with a thioredoxin fold containing a single active cysteine" | sequence and structure analysis / publication linked to PDB entry |
| PF07997 | Protein of unknown function (DUF1694) | B | 83 | 2ohw | Bacillus chorismate mutase-like | 3 | no | | |
| PF08002 | Protein of unknown function (DUF1697) | A,B,E | 119 | 2hiy | Ferredoxin-like | 2 | no | | |
| PF08538 | Protein of unknown function (DUF1749) | B,E | 77 | 2q0x | Alpha/beta hydrolase | 4 | no | | |
| PF08680 | Protein of unknown function (DUF1779) | B | 24 | 2fpn | TBP-like | 2 | no | | |
| PF08681 | Protein of unknown function (DUF1778) | B,E,V | 277 | 1y9b | Ribbon-helix-helix | 3 | no | | |
| PF08682 | Protein of unknown function (DUF1780) | B | 19 | 1y0k | Restriction endonuclease-like | 3 | yes | probably involved in processing of nucleic acids | fold and evidence of homology |
| PF08719 | Domain of unknown function (DUF1768) | B,E,V | 206 | 2b3w | Ybia-like | 0 | no | | |

| Family id | Family description (from Pfam database) | Family is represented in these kingdoms of life (A-Archaea, B-Bacteria, E-Eukaryota, V-Viruses) | Number of family members in NR (according to Pfam) | PDB id of the first structure | Fold assignment (SCOP 1.73 nomenclature) | Relationship to previously known structures (0=new fold; 1=partially similar fold; 2=putative analog; 3=putative homolog; 4=homolog) | Is it possible to propose a hypothesis about function? (1-yes, 0-no) | Hypothesis about function (quotes from publications indicated by quotation marks) | Basis of the hypothesis about function |
|---|---|---|---|---|---|---|---|---|---|
| PF08754 | Domain of unknown function (DUF1791) | A,B,E | 243 | 1l1s | Dsrefh-like | 4 | yes | "may be involved in sulfate binding and may play a role in sulfur oxidation" | sequence and structure analysis/ publication linked to PDB entry |
| PF08768 | Domain of unknown function (DUF1794) | B,E | 239 | 2a13 | Lipocalins | 3 | no | probably binds hydrophobic ligands | fold and evidence of homology |
| PF08786 | Domain of unknown function (DUF1795) | B | 100 | 1tu1 | Mog1p/psbp-like | 4 | yes | related to a subunit of oxygen evolving system | remote homology confirmed by the structure |
| PF08796 | Protein of unknown function (DUF1797) | B | 70 | 2ffg | Ykuj-like | 1 | no | | |
| PF08807 | Bacterial domain of unknown function (DUF1798) | B | 41 | 2ets | Four-helical up-and-down bundle | 2 | no | | |
| PF08827 | Domain of unknown function (DUF1805) | A,B | 36 | 1qw2 | Hypothetical protein Ta1206 | 0 | no | | |
| PF08830 | Protein of unknown function (DUF1806) | B | 43 | 1njh | Hypothetical protein yojf; some similarity to N-terminal domain of MutM-like DNA repair proteins | 1 | no | | |
| PF08837 | Protein of unknown function (DUF1810) | B,E | 83 | 2jek | Rv1873-like | 0 | no | unknown | unknown / publication linked to PDB entry |
| PF08838 | Protein of unknown function (DUF1811) | B | 39 | 1sf9 | SH3-like barrel | 3 | no | | |
| PF08848 | Domain of unknown function (DUF1818) | B | 145 | 2it9 | Ssdna-binding transcriptional regulator domain | 2 | yes | possibly transcriptional regulator | fold |
| PF08851 | Domain of unknown function (DUF1821) | B | 26 | 2plg | Secretion chaperone-like | 4 | no | | |
| PF08854 | Domain of unknown function (DUF1824) | B,E | 135 | 2q22 | Fwde/GAPDH domain-like | 2 | no | | |
| PF08860 | Domain of unknown function (DUF1827) | B | 50 | 2qzi | FAD-linked reductases, C-terminal domain | 2 | yes | possible oxidoreductase | fold |
| PF08866 | Domain of unknown function (DUF1831) | B | 58 | 2iay | TBP-like | 3 | no | | |
| PF08921 | Domain of unknown function (DUF1904) | B | 35 | 1u9d | Tautomerase/MIF | 4 | no | | |
| PF08922 | Domain of unknown function (DUF1905) | A,B | 84 | 2d9r | Double-split beta-barrel | 2 | yes | probably binds DNA | fold |
| PF08924 | Domain of unknown function (DUF1906) | B | 64 | 1sfs | TIM beta/alpha-barrel | 3 | no | | |
| PF08927 | Domain of unknown function (DUF1909) | E | 141 | 1wvk | Expressed protein At2g23090/F21P2 4.15 | 0 | no | | |
| PF08929 | Domain of unknown function (DUF1911) | B | 37 | 2fef | PA2201 C-terminal domain-like | 0 | no | | |
| PF08930 | Domain of unknown function (DUF1912) | B | 41 | 1z0p | Long alpha-hairpin | 2 | no | | |
| PF08933 | Domain of unknown function (DUF1864) | B | 61 | 1zee | Indolic compounds 2,3-dioxygenase-like | 1 | yes | oxidoreductase; putative 2,3-Dioxygenase; HEM in the core | fold, ligands |

| Family id | Family description (from Pfam database) | Family is represented in these kingdoms of life (A-Archaea, B-Bacteria, E-Eukaryota, V-Viruses) | Number of family members in NR (according to Pfam) | PDB id of the first structure | Fold assignment (SCOP 1.73 nomenclature) | Relationship to previously known structures (0=new fold; 1=partially similar fold; 2=putative analog; 3=putative homolog; 4=homolog) | Is it possible to propose a hypothesis about function? (1-yes, 0-no) | Hypothesis about function (quotes from publications indicated by quotation marks) | Basis of the hypothesis about function |
|---|---|---|---|---|---|---|---|---|---|
| PF08939 | Domain of unknown function (DUF1917) | A,B,E | 35 | 1ztp | Eif4e-like | 4 | yes | "may be involved in a biochemical process that requires recognition of nucleic acids" | sequence and structure analysis / publication linked to PDB entry |
| PF08940 | Domain of unknown function (DUF1918) | B | 40 | 2a7y | SH3-like barrel | 3 | yes | "may play a biochemical role in starvation survival and the heat shock response" | experiment / publication linked to PDB entry |
| PF08942 | Domain of unknown function (DUF1919) | B | 28 | 2g6t | CAC2185-like | 0 | no | | |
| PF08950 | Protein of unknown function (DUF1861) | A,B,E | 41 | 2b4w | 5-bladed beta-propeller | 4 | yes | probably involved in sugar binding or nucleotide hydrolysis | remote homology confirmed by the structure |
| PF08956 | Domain of unknown function (DUF1869) | B | 26 | 1nei | Hypothetical protein yoag | 1 | no | | |
| PF08958 | Domain of unknown function (DUF1871) | B | 16 | 1u84 | Yuge-like | 1 | no | | |
| PF08962 | Domain of unknown function (DUF1876) | B | 28 | 2fgg | Dsrbd-like | 2 | no | | |
| PF08963 | Protein of unknown function (DUF1878) | B | 17 | 1sed | Hypothetical protein yhai; some similarity to globin-like fold | 0 | no | | |
| PF08965 | Domain of unknown function (DUF1870) | B | 19 | 1s4k | Lambda repressor-like DNA-binding domains | 3 | yes | probably binds DNA, may be involved in regulation | fold and evidence of homology |
| PF08966 | Domain of unknown function (DUF1882) | B | 16 | 2atz | Prim-pol domain | 3 | no | | |
| PF08968 | Domain of unknown function (DUF1885) | B | 11 | 1t6a | TBP-like | 2 | no | | |
| PF08973 | Domain of unknown function (DUF1893) | B | 12 | 1vk9 | Cytidine deaminase-like | 2 | yes | a large UNL at one end of protein (may be active site); ADP-Ribosylated protein?, probably involved in metabolism of nucleotides | fold, ligands |
| PF08974 | Domain of unknown function (DUF1877) | B | 34 | 1ryl | Hypothetical protein yfbm | 0 | no | | |
| PF08975 | Domain of unknown function (DUF1868) | B,E | 105 | 2fsq | Ligt-like | 3 | yes | probably binds RNA | fold and evidence of homology |
| PF08980 | Domain of unknown function (DUF1883) | B | 28 | 2b1y | Atu1913-like | 0 | no | | |
| PF08981 | Domain of unknown function (DUF1867) | A,B | 30 | 1t57 | Pyruvate kinase C-terminal domain-like | 3 | no | FMN next to UNL; may be biologically significant | |
| PF08982 | Domain of unknown function (DUF1857) | B,E | 53 | 2ffs | TBP-like | 4 | no | | |
| PF08984 | Domain of unknown function (DUF1858) | A,B | 109 | 2fi0 | SP0561-like | 0 | no | | |
| PF08985 | Domain of unknown function (DUF1888) | B | 15 | 2ai4 | Cupredoxin-like | 3 | no | | |
| PF08986 | Domain of unknown function (DUF1889) | B | 23 | 2es9 | Yoac-like; some similarity to KaiA/RbsU domain fold | 0 | no | | |
| PF08987 | Protein of unknown function (DUF1892) | E | 9 | 1n6z | Hypothetical protein Yml108w | 0 | no | unknown | sequence and structure analysis / publication linked to PDB entry |

| Family id | Family description (from Pfam database) | Family is represented in these kingdoms of life (A-Archaea, B-Bacteria, E-Eukaryota, V-Viruses) | Number of family members in NR (according to Pfam) | PDB id of the first structure | Fold assignment (SCOP 1.73 nomenclature) | Relationship to previously known structures (0=new fold; 1=partially similar fold; 2=putative analog; 3=putative homolog; 4=homolog) | Is it possible to propose a hypothesis about function? (1-yes, 0-no) | Hypothesis about function (quotes from publications indicated by quotation marks) | Basis of the hypothesis about function |
|---|---|---|---|---|---|---|---|---|---|
| PF08989 | Domain of unknown function (DUF1896) | B | 9 | 2apl | PG0816-like | 1 | no | | |
| PF09001 | Domain of unknown function (DUF1890) | A | 17 | 1kjn | Hypothetical protein MTH777 (MT0777); similar to NAD(P)-binding Rossmann-fold domains | 1 | no | | |
| PF09002 | Domain of unknown function (DUF1887) | A,B | 23 | 1xmx | Restriction endonuclease-like | 4 | yes | probably involved in processing of nucleic acids | remote homology confirmed by the structure |
| PF09012 | Protein of unknown function (DUF1920) | B | 82 | 1xn7 | DNA/RNA-binding 3-helical bundle | 3 | yes | probably binds nucleic acids | fold and evidence of homology |
| PF09082 | Domain of unknown function (DUF1922) | A | 3 | 1gh9 | Rubredoxin-like | 2 | no | | |
| PF09123 | Domain of unknown function (DUF1931) | A,B | 15 | 1r4v | Histone-fold | 4 | yes | "possibly a DNA-binding protein that is involved in DNA packaging" | sequence and structure analysis / publication linked to PDB entry |
| PF09130 | Domain of unknown function (DUF1932) | A,B,E | 139 | 1i36 | 6-phosphogluconate dehydrogenase C-terminal domain-like | 3 | yes | dehydrogenase | fold and evidence of homology, ligand |
| PF09148 | Domain of unknown function (DUF1934) | B | 123 | 1r0u | Lipocalins | 2 | yes | probably binds hydrophobic ligands | fold |
| PF09149 | Domain of unknown function (DUF1935) | E | 120 | 1r75 | Unnamed hypothetical protein | 1 | no | | |
| PF09151 | Domain of unknown function (DUF1936) | A | 2 | 1pvm | Rubredoxin-like | 3 | yes | "electron transport protein" | experiment / publication linked to PDB entry |
| PF09152 | Domain of unknown function (DUF1937) | B | 9 | 1t1j | Flavodoxin-like | 4 | no | | |
| PF09155 | Domain of unknown function (DUF1940) | A | 3 | 1nig | Ferritin-like | 2 | yes | "probably involved in cobalamin biosyntheses" | sequence and structure analysis / publication linked to PDB entry |
| PF09167 | Domain of unknown function (DUF1942) | B | 41 | 1lmi | Immunoglobulin-like beta-sandwich | 2 | yes | "structural similarity suggests that MPT63 could be involved in cell-host interactions to facilitate endocytosis/phagocytosis" | sequence and structure analysis / publication linked to PDB entry |
| PF09171 | Domain of unknown function (DUF1886) | A | 22 | 1xg7 | DNA-glycosylase | 2 | yes | probably binds nucleic acids | fold |
| PF09179 | Domain of unknown function (DUF1946) | B | 503 | 1ni5 | Two domains: Phet/tils domain and MesJ substrate recognition domain-like | 3 | no | | |
| PF09183 | Domain of unknown function (DUF1947) | A | 7 | 1q7h | PUA domain-like | 3 | yes | probably binds RNA | fold and evidence of homology |
| PF09185 | Domain of unknown function (DUF1948) | B | 2 | 1q8c | Nusb-like | 0 | yes | "MG027 is a member of the NusB family which plays a role in antitermination processes" | sequence and structure analysis / publication linked to PDB entry |
| PF09187 | Domain of unknown function(DUF1950) | E | 8 | 1vk5 | Hypothetical protein At3g22680 | 0 | no | unknown | unknown / publication linked to PDB entry |
| PF09188 | Domain of unknown function (DUF1951) | B | 2 | 1tm9 | Hypothetical protein MG354 | 0 | no | | |

| Family id | Family description (from Pfam database) | Family is represented in these kingdoms of life (A-Archaea, B-Bacteria, E-Eukaryota, V-Viruses) | Number of family members in NR (according to Pfam) | PDB id of the first structure | Fold assignment (SCOP 1.73 nomenclature) | Relationship to previously known structures (0=new fold; 1=partially similar fold; 2=putative analog; 3=putative homolog; 4=homolog) | Is it possible to propose a hypothesis about function? (1-yes, 0-no) | Hypothesis about function (quotes from publications indicated by quotation marks) | Basis of the hypothesis about function |
|---|---|---|---|---|---|---|---|---|---|
| PF09209 | Domain of unknown function (DUF1956) | B | 113 | 1t33 | Two domains: DNA/RNA-binding 3-helical bundle and Tetracyclin repressor-like, C-terminal domain | 4 | yes | probably binds nucleic acids | remote homology confirmed by the structure |
| PF09211 | Domain of unknown function (DUF1958) | B | 32 | 1tvf | penicillin-binding protein associated domain | 2 | no | | |
| PF09218 | Domain of unknown function (DUF1959) | A | 10 | 1nxh | Hypothetical protein MTH393 | 0 | no | | |
| PF09224 | Domain of unknown function (DUF1961) | B | 11 | 1oq1 | Concanavalin A-like lectins/glucanases | 3 | no | | |
| PF09234 | Domain of unknown function (DUF1963) | B,E | 82 | 1pv5 | Hypothetical protein ywqg | 0 | no | | |
| PF09351 | Domain of unknown function (DUF1993) | B,E | 255 | 2oqm | Dinb/yfit-like putative metalloenzymes | 4 | yes | probably binds nucleic acids, possibily damage inducible proteins | remote homology confirmed by the structure |
| PF09391 | Protein of unknown function (DUF2000) | B | 84 | 2gax | Peptidyl-trna hydrolase II | 4 | yes | putative peptidyl-trna hydrolase II | remote homology confirmed by the structure |
| PF09393 | Protein of unknown function (DUF2001) | B,V | 33 | 2guj | Hcp1-like | 3 | no | | |
| PF09400 | Protein of unknown function (DUF2002) | B | 43 | 2g7j | Mota C-terminal domain-like | 2 | yes | probably binds DNA, possibly a transcription factor | fold |
| PF09407 | Protein of unknown function (DUF2005) | B | 8 | 1zel | Rv2827c C-terminal domain-like | 0 | yes | probably binds nucleic acids | structure - N-terminal domain has fold of DNA/RNA-binding 3-helical bundle |
| PF09410 | Proteins of unknown function (DUF2006) | A,B,E | 415 | 2ich | Streptavidin-like | 3 | no | | |
| PF09413 | Protein of unknown function (DUF2007) | B | 121 | 2hfv | Ferredoxin-like | 3 | no | | |
| PF09449 | Domain of unknown function (DUF2020) | B | 7 | 2i8g | TM1622-like | 0 | no | | |
| PF09450 | Domain of unknown function (DUF2019) | B | 11 | 2i9c | Alpha/alpha superhelix | 2 | no | | |
| PF09630 | Domain of unknown function (DUF2024) | A,B | 50 | 2hfq | NE1680-like; some similarity to N-terminal domain of MutM-like DNA repair proteins | 1 | no | | |
| PF09633 | Protein of unknown function (DUF2023) | A,B | 16 | 2guk | PG1857-like | 1 | no | | |
| PF09634 | Protein of unknown function (DUF2025) | B | 12 | 2hg6 | PA1123-like | 0 | no | | |
| PF09640 | Domain of unknown function (DUF2027) | B | 7 | 2huh | Immunoglobulin-like beta-sandwich | 3 | no | | |
| PF09641 | Protein of unknown function (DUF2026) | B | 2 | 2hly | Cysteine proteinases | 3 | yes | probably involved in cleavage of peptide or ester bond | fold and evidence of homology |
